# Assessment of temporal functional changes and miRNA profiling of human iPSC-derived cardiomyocytes

**DOI:** 10.1038/s41598-019-49653-5

**Published:** 2019-09-12

**Authors:** Naresh Kumar, Julie A. Dougherty, Heather R. Manring, Ibrahim Elmadbouh, Muhamad Mergaye, Andras Czirok, Dona Greta Isai, Andriy E. Belevych, Lianbo Yu, Paul M. L. Janssen, Paolo Fadda, Sandor Gyorke, Maegen A. Ackermann, Mark G. Angelos, Mahmood Khan

**Affiliations:** 1 Department of Emergency Medicine, Dorothy M. Davis Heart Lung and Research Institute, The Ohio State University Wexner Medical Center, Columbus, OH USA; 2 Department of Physiology and Cell Biology, The Ohio State University Wexner Medical Center, Columbus, OH USA; 3 Center for Biostatistics, College of Medicine, The Ohio State University Wexner Medical Center, Columbus, OH USA; 4 Comprehensive Cancer Center, The Ohio State University Wexner Medical Center, Columbus, OH USA; 5 Department of Anatomy and Cell Biology, University of Kansas Medical Center, Kansas City, KS USA

**Keywords:** Cardiovascular biology, Heart stem cells, Induced pluripotent stem cells

## Abstract

Human induced pluripotent stem cell-derived cardiomyocytes (hiPSC-CMs) have been developed for cardiac cell transplantation studies more than a decade ago. In order to establish the hiPSC-CM-based platform as an autologous source for cardiac repair and drug toxicity, it is vital to understand the functionality of cardiomyocytes. Therefore, the goal of this study was to assess functional physiology, ultrastructural morphology, gene expression, and microRNA (miRNA) profiling at Wk-1, Wk-2 & Wk-4 in hiPSC-CMs *in vitro*. Functional assessment of hiPSC-CMs was determined by multielectrode array (MEA), Ca^2+^ cycling and particle image velocimetry (PIV). Results demonstrated that Wk-4 cardiomyocytes showed enhanced synchronization and maturation as compared to Wk-1 & Wk-2. Furthermore, ultrastructural morphology of Wk-4 cardiomyocytes closely mimicked the non-failing (NF) adult human heart. Additionally, modulation of cardiac genes, cell cycle genes, and pluripotency markers were analyzed by real-time PCR and compared with NF human heart. Increasing expression of fatty acid oxidation enzymes at Wk-4 supported the switching to lipid metabolism. Differential regulation of 12 miRNAs was observed in Wk-1 vs Wk-4 cardiomyocytes. Overall, this study demonstrated that Wk-4 hiPSC-CMs showed improved functional, metabolic and ultrastructural maturation, which could play a crucial role in optimizing timing for cell transplantation studies and drug screening.

## Introduction

The discoveries in the field of human induced pluripotent stem cells (hiPSCs) have opened a novel window for stem cell therapy^1–4^. It has also demonstrated the capability of hiPSCs to differentiate into functional cardiomyocytes^1,4–8^. In recent years, stem cell therapy has shown a great potential to repair damaged heart tissue following myocardial infarction (MI)^5,9^. Furthermore, human induced pluripotent stem cell-derived cardiomyocytes (hiPSC-CMs) transplantation might serve as a viable option for cardiac regeneration^5,9,10^. Interestingly, hiPSC-CMs could provide an autologous cell source, which eliminates the possibility of cell rejection by the host immune system at the site of MI^11^. However, the cardiomyocytes derived either from pluripotent stem cells or reprogrammed somatic cells are immature in nature and resemble fetal cardiomyocytes as opposed to adult cardiomyocytes^12,13^. There are characteristic differences in morphology, gene expression, calcium signaling, and proliferation between immature and mature adult cardiomyocytes, which might impact their cardiac regeneration potential^14^.

Several studies have demonstrated time-course maturation of hiPSC-derived cardiomyocytes *in vitro* and identified changes in ultrastructural morphological characteristics, calcium transients, and cardiac gene expression^12,15,16^. A recent study by Lundy *et al*. performed a comparison of human embryonic stem cell-derived cardiomyocytes (hESC-CMs) versus hiPSC-CMs. However, the functional assessment in this study was mostly driven towards hESC-CMs^17^. Similarly, Ivashchenko *et al*. reported that hiPSC-CMs are immature cardiomyocyte-like cells and attain a functional mature phenotype during culture^18^. Maturation of hiPSC-CMs was reported in culture via electrical stimulation and structural changes were compared with human heart^19^. However, none of the aforementioned studies performed a combination of multielectrode array (MEA), particle image velocimetry (PIV), miRNAs and cardiac gene expression analysis, when compared to NF adult human heart. Therefore, the goal of this study was to perform a time-course assessment of functional maturation, ultrastructural characterization, metabolism, gene expression, and miRNAs profiling in hiPSC-CMs cultured for up to four weeks *in vitro*. To the best of our knowledge, we are the first to investigate and identify novel miRNAs modulated during prolonged culture. MEA data exhibited enhanced synchronization and maturation of cardiomyocytes in prolonged cultured cardiomyocytes. These findings were further strengthened by Ca^2+^ cycling and PIV data. Ultrastructural morphology of prolonged culture cardiomyocytes (Wk-4) mimicked the ultrastructural myofibril organization of NF adult human heart tissue. Finally, differential regulation of miRNAs, cardiac and pluripotency related genes, cell cycle gene expression, and metabolic maturation were observed in Wk-4 cardiomyocytes. Overall, understanding the functional and ultrastructural maturation of hiPSC-CMs could have several advantages, as follows: 1) optimization of the cell culture timing for transplanting studies *in vivo* after an MI; 2) utilization of these cells for cardiac patch transplantation studies, and 3) creating a more physiologically relevant platform for drug screening and disease modeling.

## Results

### Functional analysis of cardiomyocytes on MEA system

**Figure 1:**
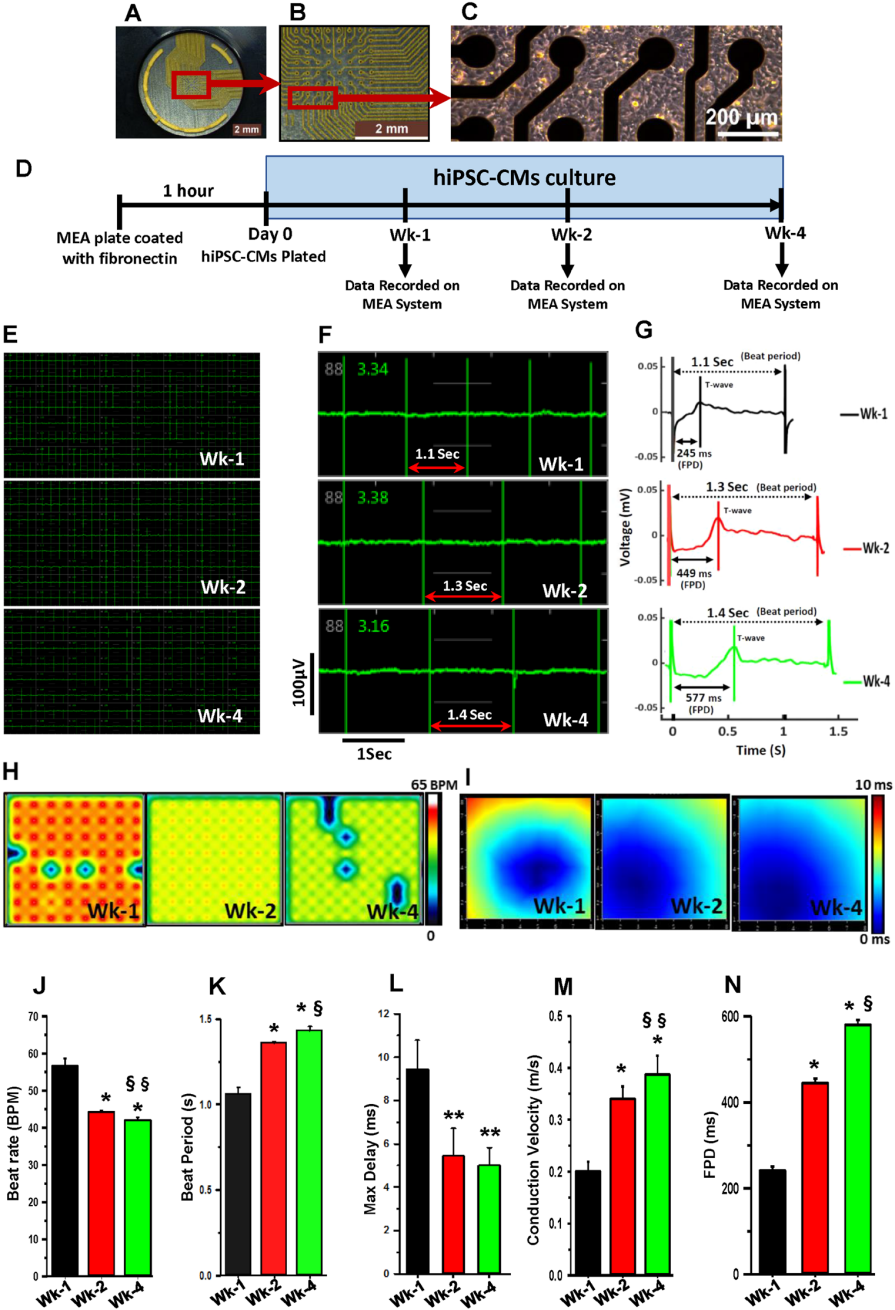
Functional analysis of cardiomyocytes on multielectrode array (MEA) system. (**A**) One well of an MEA 6 well plate consisting of 64 electrodes. (**B**) Magnified view of MEA electrodes. (**C**) Phase-contrast image of hiPSC-CMs cultured on sterile MEA plate for four weeks. (**D**) Experimental design of the study. The continuous waveform on 64 electrodes of a single well (**E**) and on one electrode (**F**). Four wells (n = 4) were selected to acquire data (64 electrodes/well). (**G**) The cardiac beat detector interprets continuous waves into cardiac beat shown in the form of a cardiac beat plot, representative image. (**H**) Long-time culture has shown changes in activity map which shows the decrease in beats per minute (BPM), representative image. (**I**) Conduction plot showing propagation delay of hiPSC-CMs cultured for Wk-1, Wk-2, and Wk-4, the blue region represents the origin of the beat (start electrode) while different colors showing propagation delay time as shown in scale bar, representative image. (**J**) Change in beat rate beat per minute (BPM) and (**K**) beat period in hiPSC-CMs cultured for Wk-1, Wk-2, and Wk-4 (s = second). (**L**) Max. Delay (Difference in beat detection time between electrodes in a well) (ms = millisecond). (**M**) Conduction velocity (m/s = meter per second) and, (**N**) Field potential duration (FPD) in hiPSC-CMs cultured for Wk-1, Wk-2, and Wk-4. (ms = millisecond) *p < 0.001 vs Wk-1, **p < 0.05 vs Wk-1, ^§^p < 0.001 vs Wk-2, ^§§^p < 0.05 vs Wk-2. Data expressed as mean ± SD (n = 4).

The functional analysis of cardiomyocytes was performed on an MEA system, which executed non-invasive data recording^20–22^. It measures real-time excitability of the cardiomyocytes in the culture plate without interfering with membrane integrity. hiPSC-CMs were cultured on MEA plates (Fig. 1A–C) and data was acquired on the same plate after 1 (Wk-1), 2 (Wk-2), and 4 (Wk-4) weeks, as described in Fig. 1D. Continuous waveform showed the contractility of hiPSC-CMs on 64 electrodes of the well (Fig. 1E). There was a prominent change in the beat period at Wk-1, Wk-2, and Wk-4 (Fig. 1F). The cardiac beat detector interpreted the continuous waveform into the cardiac beat and is shown in the form of a cardiac beat plot (Fig. 1G). The activity map (Fig. 1H) and bar diagram (Fig. 1J) showed that the beat rate decreased significantly (p < 0.001) at Wk-2 (44 ± 0.3 BPM) and Wk-4 (42 ± 0.8 BPM) as compared to Wk-1 hiPSC-CMs (57 ± 2.1 BPM). Excitingly, the MEA data analysis showed significant (p < 0.001) increases in the beat period at Wk-2 (1.36 ± 0.01 s) and Wk-4 (1.43 ± 0.03 s) as compared to Wk-1 hiPSC-CMs (1.06 ± 0.04 s) (Fig. 1K). The conduction map (Fig. 1I) showed a delay in conduction, which was significantly (p < 0.001) decreased at Wk-2 (5.44 ± 0.28 ms) and Wk-4 (5.01 ± 0.82 ms) as compared to Wk-1 hiPSC-CMs (9.46 ± 1.33 ms) (Fig. 1L). Recent studies have shown that conduction velocities vary from 0.04 m/s to 1.0 m/s in hiPSC-CMs^23,24^. In our study, Wk-2 and Wk-4 hiPSC-CMs showed a significant (p < 0.001) increase in conduction velocity (0.34 ± 0.02 m/s and 0.39 ± 0.04 m/s, respectively), when compared to the Wk-1 hiPSC-CMs (0.20 ± 0.02 m/s) (Fig. 1M). Interestingly, the increased conduction velocity at Wk-4 was comparable to the conduction velocity of the adult human heart (0.5 m/s)^25^. Additionally, field potential duration **(**FPD) significantly (p < 0.001) increased at Wk-2 (445 ± 10 ms) and Wk-4 hiPSC-CMs (580 ± 11 ms) as compared to Wk-1 hiPSC-CMs (241 ± 10 ms) (Fig. 1N). These data demonstrate the increased electrical functionality of the cells over time in culture.

### Measurement of spontaneous contractile activity in hiPSC-CMs

**Figure 2:**
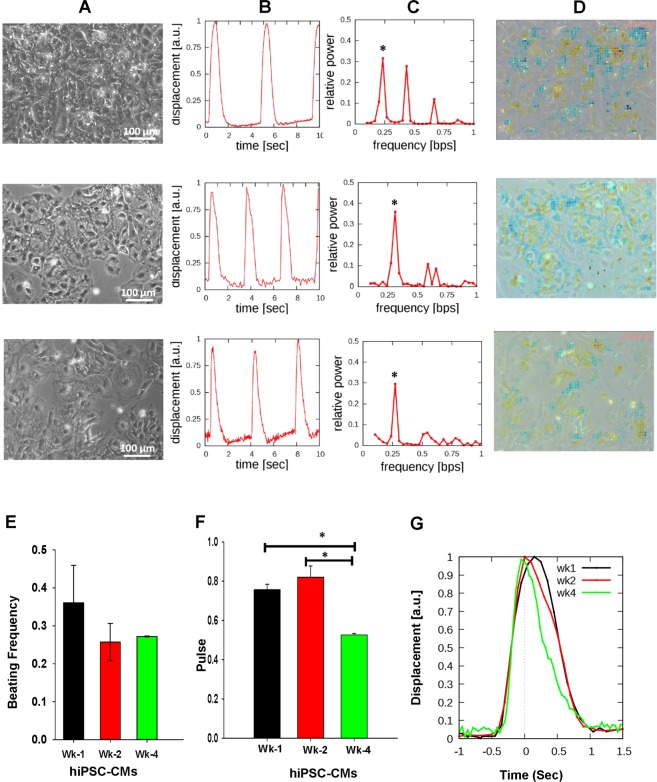
The contractile waveform analysis by particle image velocimetry (PIV) showing quicker contraction in mature cardiomyocytes. (**A**) Phase contrast microscopic images of differentiated and beating cardiomyocytes visualized by high framerate PIV. (**B**) The spontaneous beat pattern (PIV-derived displacements, measured relative to a stationary reference state and averaged over a 100-pixel radius large field of view) indicates that cardiomyocyte contractility is periodic with a steady waveform. (**C**) Fourier power spectra of beat patterns show the dominant beat frequency as a peak (asterisks). (**D**) Contractility analysis overlayed onto the original phase-contrast image. Contractile behavior is indicated by warmer colors, while cooler colors indicate local expansion of the cell collective. (**E**) Evolution of the spontaneous beating frequency during a four-week interval, obtained from recordings of Wk-1, Wk-2, and Wk-4 hiPSC-CMs. The beat frequency is stable in the investigated time period; the differences are not statistically significant and reflect a wide distribution of beat frequencies in various cultures. (**F**) The length of the contractile events is decreasing with culture age (p < 0.05). (**G**) Average contractile profiles indicate progressively quicker contraction and a prolonged relaxation as cultures mature *in vitro*. Black, red and green colors indicate progressively older (Wk-1, Wk-2, and Wk-4 hiPSC-CMs) cultures, respectively.

The functionality of hiPSC-CMs was studied by high framerate PIV, carried out at Wk-1, Wk-2, and Wk-4 at room temperature *in vitro* (Fig. 2A). Image analysis of the image sequences reveals a spontaneous beat pattern that is stable in time and devoid of arrhythmias (Fig. 2B,C). A computational measure of contractility indicates a beating activity that is non-uniformly distributed: the contraction of the same subset of cells is accommodated by the expansion of their neighbors in each cycle (Fig. 2D). The contraction of the beating cells, however, remains synchronous in time during the entire observation period (Supplemental Fig. S1). The mean beating frequency is 0.3 Hz (18 BPM), which does not change significantly after the onset of contractions (Fig. 2C). Although the frequency remains stable (Fig. 2E), the duration and intensity of contraction changes over time (Fig. 2F,G). The contraction shape resembles a Gaussian curve for younger cultures (the contraction and relaxation phases are similar); while mature cultures exhibit more sudden contractions and prolonged relaxation (Fig. 2G). Our contractility measurements also indicate higher values for mature cultures (peaks reaching 1.5/sec in Wk-1 hiPSC-CMs, while reaching 3/sec in Wk-4 hiPSC-CMs (see Supplemental Fig. S1). Overall, these results demonstrate improvement in contractility over time in culture.

### Measurement of Ca^2+^ transient during maturation in hiPSC-CMs

**Figure 3:**
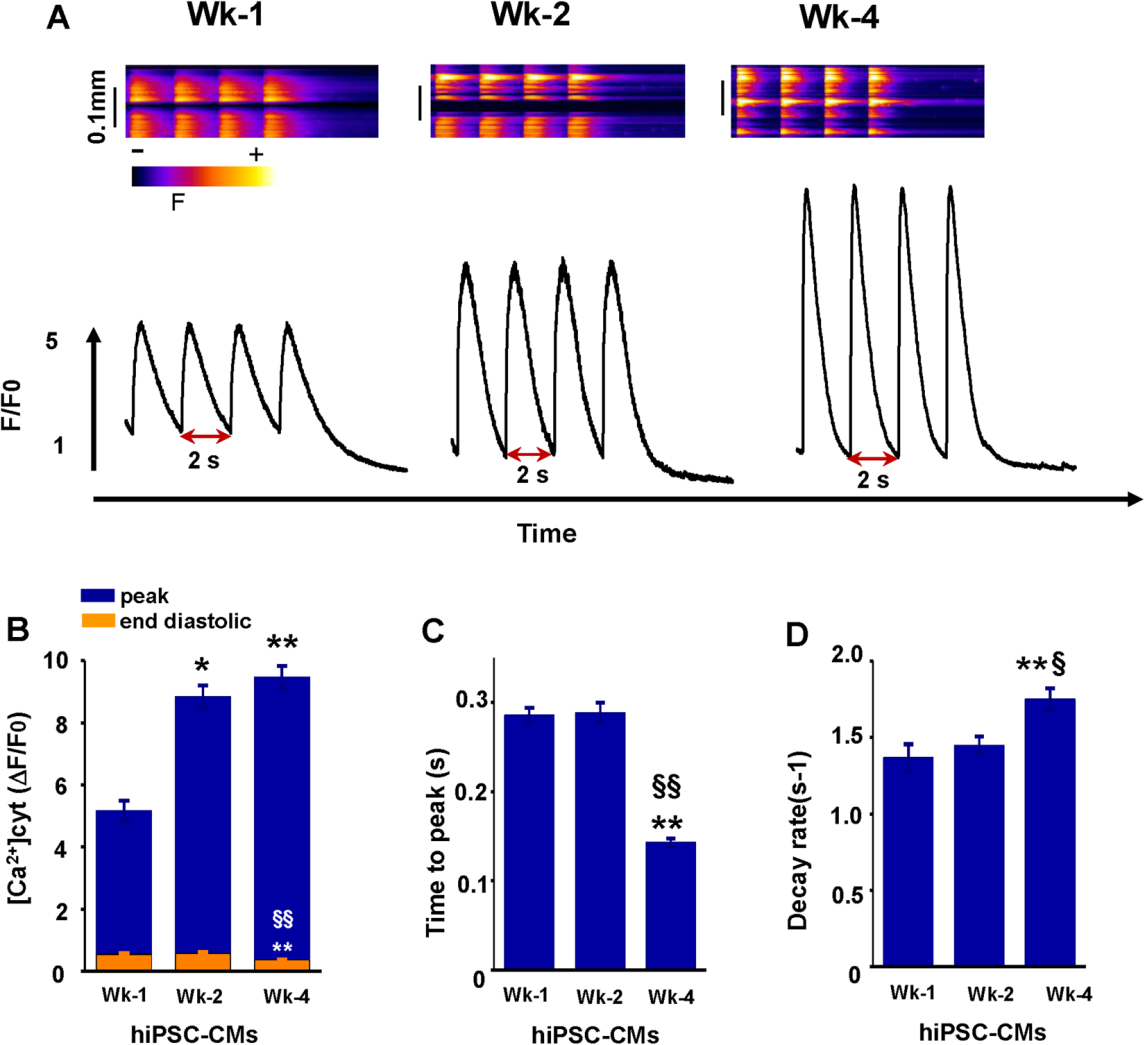
Time-dependent changes in Ca^2+^ transient (single-cell analysis) during maturation. (**A**) Representative line scan images with corresponding spatially averaged profiles of cytosolic Ca^2+^ transients evoked by electrical field stimulation at 0.5 Hz in cells cultured for Wk-1, Wk-2, and Wk-4, respectively. (**B**) Average values for systolic and end decay of Ca^2+^ cyt. (**C**) Time to Ca^2+^ transient peaks. (**D**) Rate of decay of Ca^2+^ transients recorded in Wk-1 (n = 51–59), Wk-2 (n = 41–46), and Wk-4 (n = 71–76), where ‘n’ indicates number of cells. *p < 0.05 vs Wk-1, **p < 0.01 vs Wk-1, ^§^p < 0.05 vs Wk-2, ^§§^p < 0.001 vs Wk-2 (t-tests with Bonferroni correction).

Ca^2+^ cycling of cardiomyocytes was studied in Wk-1, Wk-2, and Wk-4 hiPSC-CMs. hiPSC-CMs were loaded with Ca^2+^-sensitive dye fluo-3 and Ca^2+^ transients were evoked by electrical field stimulation at 0.5 Hz. As illustrated in Fig. 3A,B, Ca^2+^ transient amplitude increased progressively with the duration of cell culture. Significant acceleration of two different Ca^2+^ transient kinetics, time to peak (Fig. 3C) and decay rate (Fig. 3D) were observed in Wk-4 hiPSC-CMs when compared to Wk-1 and Wk-2 hiPSC-CMs. These kinetic changes were associated with reduced end-diastolic Ca^2+^ level in Wk-4 cardiomyocytes (Fig. 3A,B). These results demonstrate the augmentation of calcium handling capabilities of hiPSC-CMs over time.

### Metabolic maturation of cardiomyocytes

**Figure 4:**
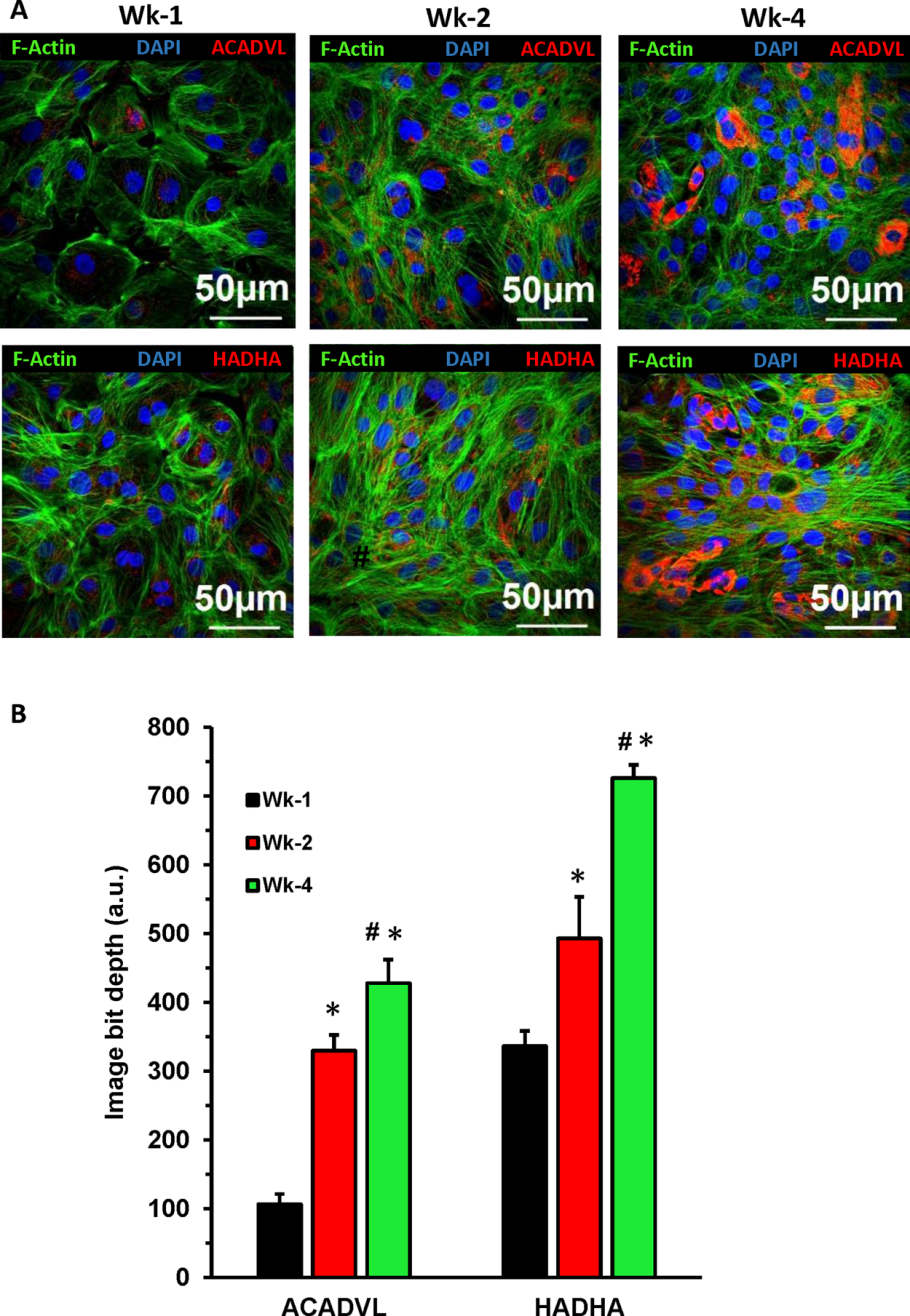
Immunofluorescence microscopy showing the metabolic maturation of cardiomyocytes by switching towards fatty acids β-oxidation pathways. (**A**) Cells were cultured for Wk-1, Wk-2, and Wk-4 and then immunostained with anti-ACADVL and anti-HADHA. (**B**) An image depth comparison showing a significantly increased level of ACADVL and HADHA in prolonged cultured hiPSC-CMs as compared to short-time cultured. The image depth was measured on Olympus FLUOVIEW Ver. 4.2a Viewer. Data expressed as mean ± SD, n = 4, *p < 0.05 vs Wk-1, ^#^p < 0.05 vs Wk-2.

Cardiomyocytes switch from glycolytic pathways to fatty acids β-oxidation (FAO) pathways as they mature^26–28^. The metabolic maturation of cardiomyocytes was studied by assaying the expression of two vital enzymes of FAO pathways: acyl-CoA dehydrogenase very long chain, (ACADVL, EC 1.3.99.13) and α-subunit of long-chain 3-hydroxyacyl coenzyme A dehydrogenase (HADHA, EC 1.1.1.211). Confocal microscopy (Fig. 4A) showed that the expression of ACADVL and HADHA was significantly (p < 0.05) increased in Wk-4 as compared to Wk-1 and Wk-2 hiPSC-CMs (Fig. 4B, Supplemental Fig. S2).

### Analysis of hiPSC-CMs maturity via Transmission Electron Microscopy (TEM)

**Figure 5:**
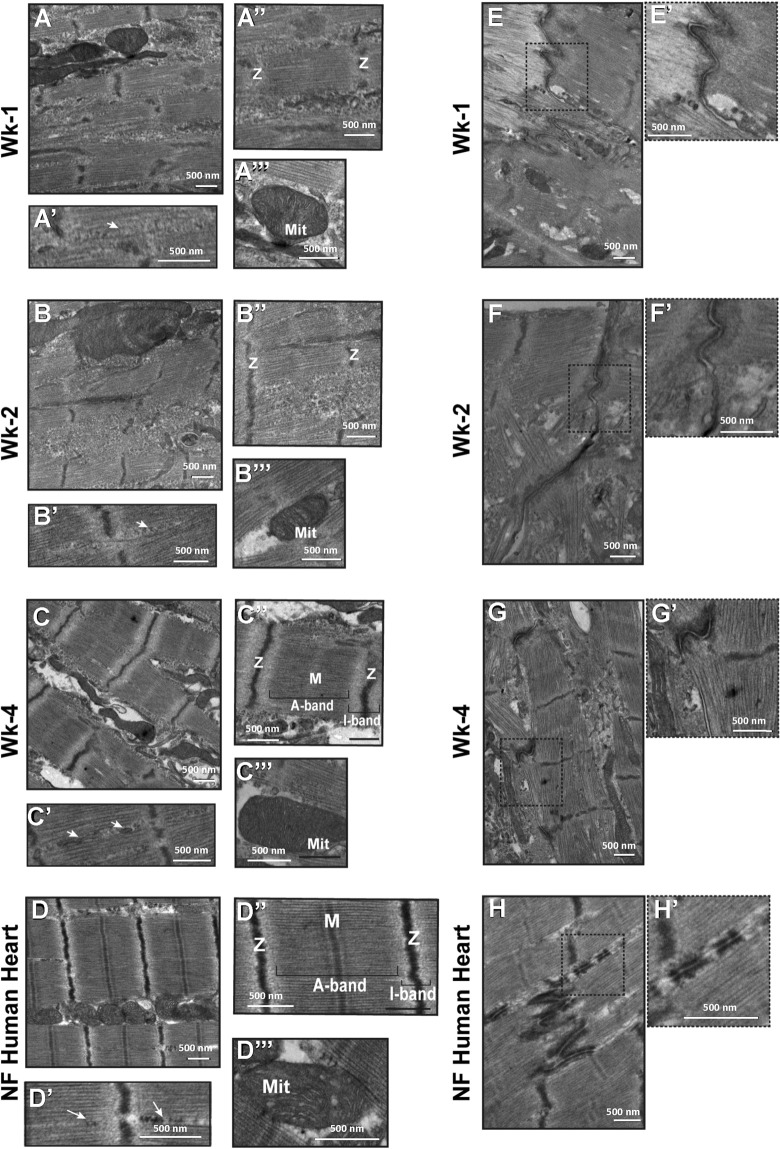
Refinement of ultrastructural features of hiPSC-CMs as compared to NF adult human heart. TEM illustrates overall refinement in hiPSC-CMs structure with increased cell culture time. (**A–C**) hiPSC-CMs show increased myofibril organization and cardiomyocyte structure from Wk-1 (**A**) to Wk-2 (**B**) and Wk-4 (**C**) in culture. (**A’–C’**) Internal membranes (white arrow) are punctate and disorganized at Wk-1 and Wk-2 but are organized at Wk-4, where internal membrane formation and incorporation between the myofilaments is evident. (**A”–C”**) Sarcomere structure shows refinement over the time span with hiPSC-CMs cultured at Wk-4 showing highly prominent and well-defined M-bands, I-bands, and A-bands as well as the alignment of the Z-discs. Z = Z-disc, M = M-band. (**A’”–C’”**) Mitochondria (Mit) are present in cells cultured at all three-time points. (**E**–**G**) TEM identifies intercalated discs for Wk-1, Wk-2, and Wk-4 hiPSC-CMs. Length of the representative intercalated disc is traced. (**E’**–**G’**) Increased magnification shows advanced ultrastructure of intercalated discs including the development of highly electron-dense regions, likely representative of desmosomes. (**D**) TEM sections from the left ventricle of NF adult human heart exhibit advanced myofibril organization and cardiomyocyte structure. (**D’**) Internal membranes are developed and present throughout the sarcomeres. (**D”**) Sarcomeres show distinct separation into Z-disk, M-band, A-band, and I-band with mitochondria present with visible cristae (**D’”**). (**H**) The intercalated disc is well defined in human heart tissue. The length of the intercalated disc is traced. (H’) Human heart tissue shows advanced ultrastructural features of the intercalated disc including the presence of desmosomes. Scale bar, 500 nm.

The ultrastructural TEM analysis of cardiomyocytes cultured for different weeks showed significant differences. The Wk-1, Wk-2, and Wk-4 hiPSC-CMs have regions of both refinement and disorganization. At Wk-1 the refined regions were minimal compared to the disorganized regions, however, at Wk-4 the disorganized regions were minimal compared to the refined regions (Supplemental Fig. S3). Overall, the Wk-4 hiPSC-CMs showed increased myofibril organization, and more defined and organized Z-discs than Wk-1 and Wk-2 hiPSC-CMs (Fig. 5A–C). The myofibril organization of Wk-4 hiPSC-CMs was in accord with the myofibrils of the NF adult human heart (Fig. 5D). More Z-bodies (Zb) and stars were present in the Wk-2 hiPSC-CMs (Supplemental Fig. S4). In Wk-1 hiPSC-CMs, the presence of the A-bands, I-bands, Z-bands, and M-bands were not noticeable (Fig. 5A’’); while in Wk-2 hiPSC-CMs, the presence of the Z-bands was noticeable but A-bands, I-bands, and M-bands were still not well-defined (Fig. 5B’’). However, in Wk-4 hiPSC-CMs, the M-bands were prominent and most sarcomeres were showing the separation of the actin and myosin into their respective A and I-bands (Fig. 5C’’), which was similar to the NF adult human heart (Fig. 5D’’). In Wk-1 hiPSC-CMs, there was little evidence of internal membranes and mostly myofilaments were present (Fig. 5A’ white arrow). On the other hand, Wk-2 cells had an empty space between the myofilaments, and internal membrane-like structures had begun to form (Fig. 5B’ white arrow). The internal membrane of Wk-1 and Wk-2 hiPSC-CMs was disorganized and punctate. Contrarily, Wk-4 hiPSC-CMs had some areas of internal membranes that were integrated between the myofilaments (Fig. 5C’ white arrow), which were similar to the NF adult human heart (Fig. 5D’ white arrow). The mitochondria of Wk-4 hiPSC-CMs were well organized within the cells (Fig. 5C’’’) and appeared larger than Wk-1 and Wk-2 hiPSC-CMs (Fig. 5A’’’,B’’’). Interestingly, Wk-4 mitochondria were more similar to the NF adult human heart, yet they were not associated with sarcomeres as seen in the NF adult human heart (Fig. 5D’’’). Intercalated discs were present in Wk-1, Wk-2, and Wk-4 hiPSC-CMs (Fig. 5E–G). Portions of the intercalated disc progressed towards a more defined electron-dense region corresponding to desmosomal structures (Fig. 5E’,F’,G’). In Wk-4 hiPSC-CMs, the intercalated disc developed the tread-step-tread layout, which is typical of human heart structures (Fig. 5H,H’). The results demonstrate that, on an ultrastructural level, Wk-4 hiPSC-CMs closely resemble NF adult heart tissue.

### Cardiac gene expression changes in hiPSC-CMs

#### Pluripotency markers

**Figure 6:**
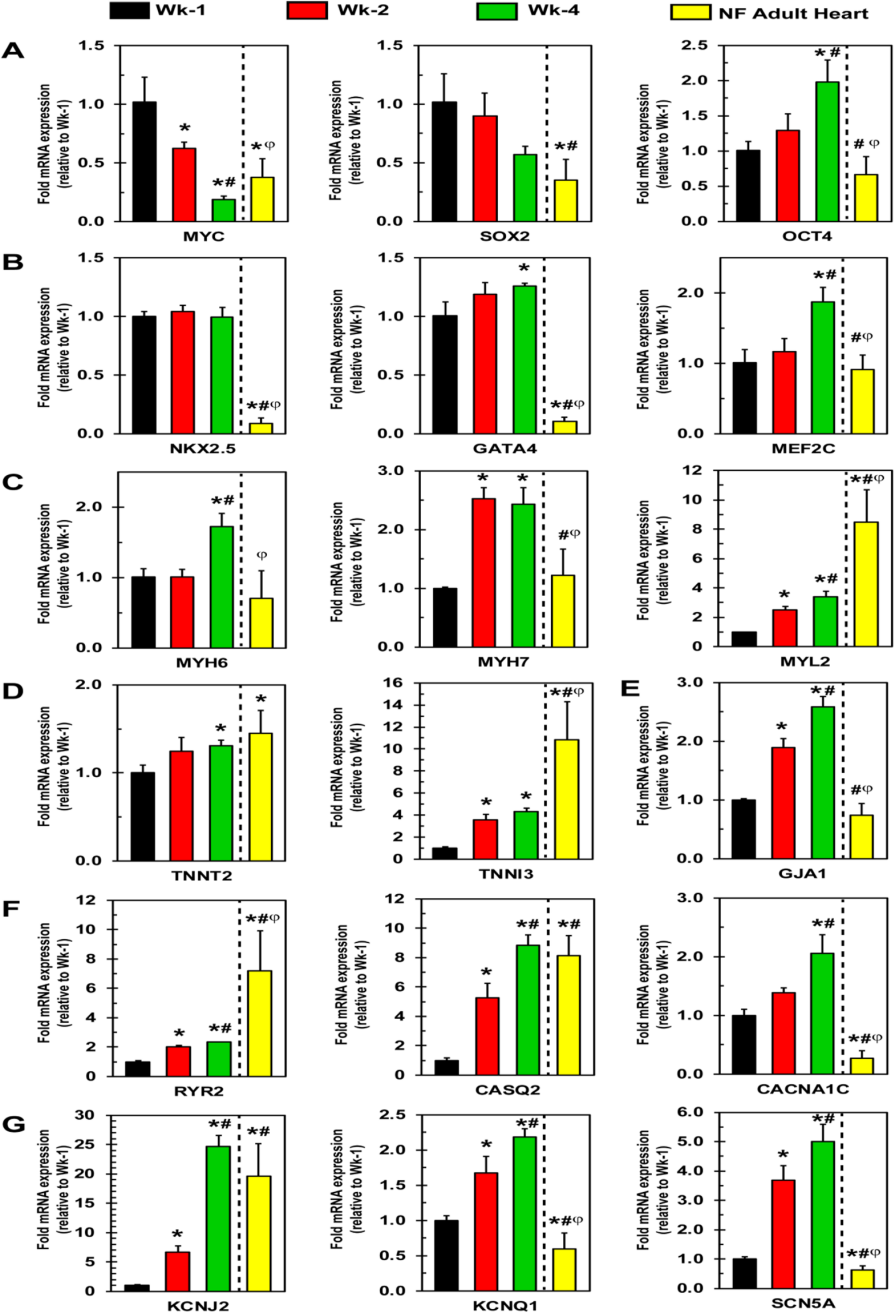
RT-qPCR analysis showing the expression of pluripotency and cardiac maturity markers in hiPSC-CMs. Cells were cultured for Wk-1, Wk-2, and Wk-4 and the result show expression of (**A**) pluripotency markers, (**B**) cardiac transcription factors, (**C**,**D**) contractile genes, (**E**) Gap junction, (**F**) Ca^2+^ handling, and (**G**) K^+^/Na^+^ channel genes. Data expressed as mean ± SD, n = 3 for cells, n = 9 for NF adult human heart, *p < 0.05 vs Wk-1, ^#^p < 0.05 vs Wk-2, ^ϕ^p < 0.05 vs Wk-4.

First, expression of pluripotency markers MYC, SOX2, and OCT4 were performed. Results showed that MYC was significantly (p = 0.003) decreased in Wk-4 cardiomyocytes as compared to Wk-1 (Fig. 6A). Alternatively, the expression level of MYC in the NF adult human heart was significantly (p = 0.016) higher than Wk-4 levels (Fig. 6A). SOX2 showed decreased expression at Wk-2 and Wk-4, although statistically insignificant. NF adult human heart SOX2 levels were significantly (p = 0.012) lower as compared to Wk-4 (Fig. 6A). Interestingly, we found that OCT4 expression increased significantly (p = 0.008) at Wk-4 as compared to Wk-1. Lastly, NF adult human heart OCT4 expression was significantly (p = 2.48e-5) lower compared to Wk-4 expression (Fig. 6A).

#### Cardiac transcription factors

Next, we tested the expression of cardiac transcription factors NKX2.5, GATA4, and MEF2C. Results showed that hiPSC-CM expression of NKX2.5 was steady for the duration of experiments (Fig. 6B). NF adult human heart NKX2.5 expression was significantly (p = 0.016) decreased as compared to all time points (Fig. 6B). GATA4 expression in hiPSC-CMs increased significantly (p = 0.023) by Wk-4 as compared to Wk-1 (Fig. 6B). Again, NF adult human heart expression was significantly decreased as compared to all time points (p = 2.97e-13 vs Wk-4) (Fig. 6B). MEF2C was increased significantly (p = 0.006) at Wk-4 as compared to Wk-1 (Fig. 6B). MEF2C expression in the NF adult human heart was found to be nearly equal to Wk-1 and significantly (p = 3.9e-5) decreased as compared to Wk-4 (Fig. 6B).

#### Cardiac contractile genes

Further, we compared the expression of contractile genes including myosins and troponins. MYH6, α heavy chain, expression increased significantly (p = 0.005) for Wk-4 as compared to Wk-1 (Fig. 6C). NF Adult human heart MYH6 expression was significantly (p = 0.002) lower than Wk-4 (Fig. 6C). Alternatively, MYH7, β heavy chain, expression significantly (p = 0.0001 and p = 0.0009, respectively) increased for Wk-2 and Wk-4 as compared to Wk-1 (Fig. 6C). Interestingly, NF adult human heart MYH7 expression levels were comparable to Wk-1 and significantly (p = 0.001) lower than Wk-4 (Fig. 6C). MYL2 encodes myosin light chain 2, which is the isoform found in the ventricles^29^. MYL2 expression increased significantly (p = 0.006 and p = 0.0003, respectively) for Wk-2 and Wk-4 as compared to Wk-1 (Fig. 6C). However, NF adult human heart expression was significantly (p = 0.003) and substantially increased as compared to Wk-4 (Fig. 6C).

Additionally, TNNT2, cardiac troponin T, increased significantly (p = 0.005) but modestly in Wk-4 as compared to Wk-1 (Fig. 6D). NF adult human heart TNNT2 expression was significantly (p = 0.018) increased as compared to Wk-1, similar to Wk-4 (Fig. 6D). TNNI3, cardiac troponin I, which is the only detectable isoform in mature human hearts^30^, showed significantly increased (p = 0.006 and p = 0.001, respectively) expression for Wk-2 and Wk-4 as compared to Wk-1 (Fig. 6D). NF adult human heart expression, however, was significantly (p = 0.01) and substantially increased as compared to Wk-4 (Fig. 6D).

#### Genes involved in gap junctions and calcium handling

Next, we analyzed expression levels for Connexin 43 and calcium handling genes. Connexin-43, encoded by GJA1, forms gap junctions to electrically couple myocytes and aid in synchronization of contraction^31^. The expression of GJA1 increased significantly (p = 0.0005 and p = 0.0001, respectively) for Wk-2 and Wk-4 as compared to Wk-1 (Fig. 6E). GJA1 expression in NF adult human heart tissue was significantly (p = 6.34e-8) decreased as compared to Wk-4 and similar to that of Wk-1 (Fig. 6E). Next, RYR2, which encodes ryanodine receptor 2 showed significantly (p = 6e-5 and p = 9e-6, respectively) increased expression at Wk-2 and Wk-4 as compared to Wk-1 (Fig. 6F). NF adult human heart tissue expression levels were significantly (p = 0.002) and considerably increased as compared to Wk-4 (Fig. 6F). CASQ2 expression increased dramatically and significantly (p = 0.016 and p = 4e-5, respectively) for Wk-2 and Wk-4 as compared to Wk-1 (Fig. 6F). NF adult human heart tissue CASQ2 expression levels were similar to Wk-4 (Fig. 6F). CACNA1C encodes a subunit of the voltage-gated calcium channel Cav1.2 present on the cell membrane^32^. Our results showed that the expression of CACNA1C significantly (p = 0.005) increased for Wk-4 as compared to Wk-1 (Fig. 6F). Interestingly, the level of CACNA1C expression in NF adult human heart tissue was dramatically and significantly (p = 5e-6) decreased as compared to Wk-4 (Fig. 6F).

#### Potassium and sodium handling genes

Furthermore, we examined the expression of  genes involved in K^+^ and Na^+^ handling that increase during maturation to facilitate the increased demand of ion handling for proper excitation-relaxation. KCNJ2, which encodes Kir2.1, expression increased significantly (p = 0.0098 and p = 2.8e-5, respectively) for Wk-2 and for Wk-4 as compared to Wk-1 (Fig. 6G). The Wk-4 expression level was similar to that of NF adult human heart tissue (Fig. 6G). Next, we tested the expression of KCNQ1, which encodes Kv7.1, and its expression increased significantly (p = 0.0085 and p = 0.0001, respectively) for Wk-2 and Wk-4 as compared to Wk-1 (Fig. 6G). NF adult human heart tissue KCNQ1 expression levels were significantly (p = 0.016 vs Wk-1) lower than all cellular levels (Fig. 6G). SCN5A, which encodes Nav1.5, expression was significantly increased (p = 0.0007 and p = 0.0066, respectively) for Wk-2 and Wk-4 as compared to Wk-1 (Fig. 6G). NF adult human heart tissue expression was significantly (p = 0.002 vs Wk-1) lower than all cellular levels (Fig. 6G). Conclusively, increased expression of these genes is the molecular basis for the increased functionality of the cells as seen by our other experiments.

### Cell Cycle Genes

**Figure 7:**
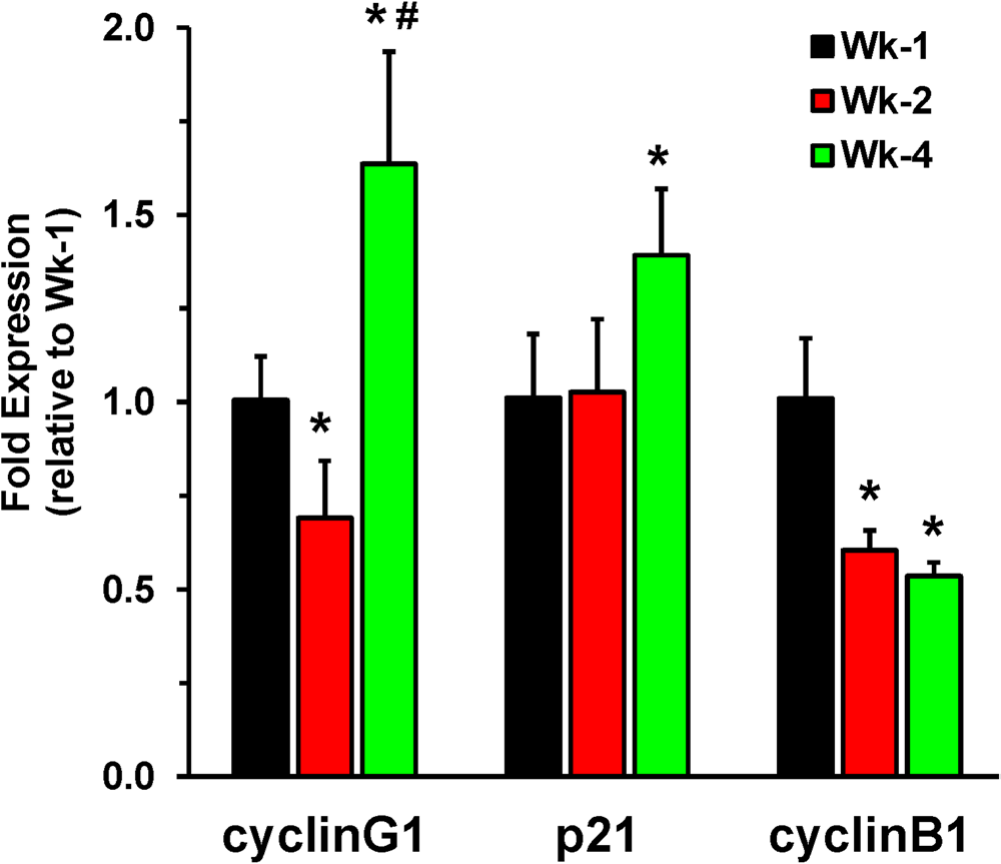
RT-qPCR analysis showing the expression of cell cycle genes in hiPSC-CMs. Cells were cultured for Wk-1, Wk-2, and Wk-4 and the results show expression of cell cycle genes. Data expressed as mean ± SD, n = 4, *p < 0.05 vs Wk-1, ^#^p < 0.05 vs Wk-2.

Cell cycle gene expression in hiPSC-CMs was performed at Wk-1, Wk-2, and Wk-4. Our results showed a significant (p = 0.008) increase in cyclinG1 expression at Wk-4, as compared to Wk-1 levels (Fig. 7). Similarly, the expression of p21 in hiPSC-CMs increased significantly (p = 0.035) at Wk-4 as compared to Wk-1. Additionally, results show significant decreases in expression of cyclinB1 at Wk-2 (p = 0.011) and Wk-4 (p = 0.008) as compared to Wk-1. Collectively, the results indicate that after 4 weeks of hiPSC-CMs culturing, they exit the cell cycle to enable polyploidization and maturation.

### miRNA profiling of hiPSC-CMs during time-course culture

**Figure 8:**
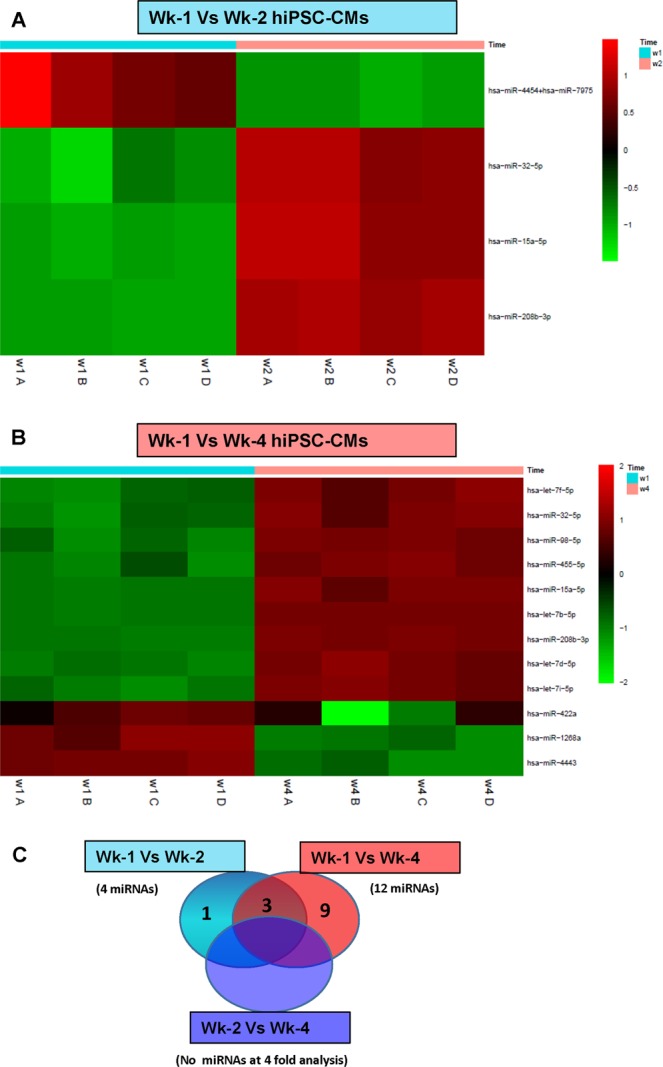
Identification of maturation-associated miRNAs in hiPSC-CMs during time-course culturing. hiPSC-CMs were cultured for Wk-1, Wk-2, and Wk-4, and the expression profiles of 800 miRNAs were analyzed. (**A**) miRNAs comparison of Wk-1 vs Wk-2 hiPSC-CMs; 4 miRNAs were differentially regulated. (**B**) miRNAs comparison of Wk-1 vs Wk-4 hiPSC-CMs; 12 miRNAs were differentially regulated. (**C**) Venn diagram showing that 3 miRNAs were common between Wk-1 vs Wk-2 hiPSC-CMs and Wk-1 vs Wk-4 hiPSC-CMs.

Since their discovery, microRNAs (miRs) have emerged as key regulators of gene expression, estimated to regulate 30% of human genes^33^. The expression profiles of 800 miRNAs were analyzed in Wk-1, Wk-2, and Wk-4 hiPSC-CMs. Four miRNAs were differentially regulated in Wk-2 hiPSC-CMs when compared to Wk-1 hiPSC-CMs (Fig. 8A). Among these miRNAs, three miRNAs were upregulated (miR-32-5p, miR−15a−5p, and miR−208b−3p) and one miRNA was downregulated (miR-4454 + miR-7975). On the other hand, when Wk-1 hiPSC-CMs were compared with Wk-4 hiPSC-CMs, twelve miRNAs were differentially regulated. Among these, nine miRNAs (let−7 f−5p, miR−32−5p, miR−98−5p, miR−455−5p, miR−15a−5p, let−7b−5p, miR−208b−3p, let−7d−5p, and let−7i−5p) were upregulated at Wk-4 while three were downregulated (miR−422a, miR−1268a, and miR−4443) (Fig. 8B). Three miRNAs were common between Wk-1 vs Wk-2 hiPSC-CMs and Wk-1 vs Wk-4 hiPSC-CMs (Fig. 8C). These data demonstrate changes in miRNA expression occur over time the hiPSC-CMs are in culture.

## Discussion

Immature cardiomyocytes are similar to fetal cardiomyocytes^34,35^. However, it is unclear what type of hiPSC-CMs (mature or immature cardiomyocytes) would be a suitable candidate for cardiac cell transplantation. In some studies, neonatal or fetal cardiomyocytes have been shown to provide a promising role in therapy in ischemic heart disease^36–38^. Additionally, in another study, immature hiPSC-CMs reported a better integration with host tissue but failed to cope with host action potential^39^. Contrarily, other studies have reported that transplantation of mature cardiomyocytes shows a higher engraftment ratio and more significant functional outcome than immature cardiomyocytes^9,40^. Therefore, maturation of hiPSC-derived cardiomyocytes *in vitro* will have a significant impact on cell transplantation studies *in vivo*, enhancing the functional outcome of transplanted cells in the ischemic myocardium.

In our study, the functional activity of hiPSC-derived cardiomyocytes was analyzed on an MEA system. Results demonstrated an increase in beat period duration and a decrease in beat rate in prolonged cultured cardiomyocytes (Wk-4 hiPSC-CMs) as compared to short-term cultured cardiomyocytes (Wk-1 hiPSC-CMs). Similarly, there was an increase in the conduction velocity and decrease in Max delay in prolonged cultured cardiomyocytes as compared to short-term cultured. Results showed that the cardiomyocytes cultured for up to four weeks exhibited more synchronized beating as compared to Wk-1 cardiomyocytes. Interestingly, the increased conduction velocity in prolonged cultured cardiomyocytes (0.4 m/s) correlated with the conduction velocity reported earlier^20^ and in the adult human heart (0.5 m/s)^25^. Therefore, the decreased delay in wave propagation and increased conduction velocity in hiPSC-CMs cultured for four weeks showed that cells were more synchronized and closely mimicking the adult human heart. The PIV-based functional analysis of cardiomyocytes showed variations in contraction pattern. Spontaneous beating activity was readily detected from Wk-1, with well-separated contractile and resting phases. The contractile cells were synchronous in time. Additionally, the contractile waveforms also reflect a gradual maturation process. Particularly, contractions became quicker and stronger in prolonged cultured cardiomyocytes. We noticed that PIV and MEA analysis showed a difference in beat rate. This variation in beat rate may be due to the differences in recording temperatures for hiPSC-CMs. Nevertheless, the analysis of both results indicate a decreased beat rate in prolonged cultured hiPSC-CMs as compared to short-time cultured. Furthermore, in accord with the MEA and PIV results, Ca^2+^ cycling data also demonstrated decreased time to peak ratio and faster decay rate in prolonged cultured cardiomyocyte indicating improvement in their functionality and maturation.

During the process of cardiomyocyte maturation, one of the major changes that takes place is the metabolic switching of energy source from glycolytic pathways to fatty acid β-oxidation pathways (FAO)^26–28^. Immunostaining analysis showed that the expression levels of FAO enzymes, ACADVL and HADHA, were significantly increased in prolonged cultured hiPSC-CMs. Thus, the prolonged cultured hiPSC-CMs were metabolically more mature as compared to short-time cultured cardiomyocytes due to greater utilization of FAO.

Ultrastructural TEM analysis results from hiPSC-CMs cultured at different weeks demonstrated significant structural differences, which can be classified into two parameters- refinement and organization. The increased myofibril organization and cardiomyocyte structure in prolonged cultured is a hallmark of the maturation of cardiomyocytes. Firstly, Z-discs were more defined and organized in Wk-4 as compared to Wk-1 or Wk-2 hiPSC-CMs. Secondly, the ultrastructural analysis was focused on the multiple bands that form sarcomeres which were prominent in Wk-4 hiPSC-CMs, with well-defined A, I, and Z-bands, along with the characteristic M-band^41^. Thirdly, TEM analysis demonstrated high variability in the number, size, development, and organization of mitochondria in cardiomyocytes cultured for different weeks. In general, the mitochondria in the Wk-4 hiPSC-CMs were more organized and larger than Wk-1 and Wk-2 hiPSC-CMs. The compactness of mitochondria is directly proportional to the ATP synthesis demand of the contractile machinery in mature cardiomyocytes^42^. The fourth facet of the ultrastructural analysis emphasized the propagation of action potentials and wave propagation. Desmosomes provide physical strength and gap junctions consisting of connexins increase cell-to-cell communication^43^. We did not observe any differences in the number of desmosomes and gap junctions in Wk-2 and Wk-4 hiPSC-CMs. However, there were differences in their level of organization. Interestingly, some of the intercalated discs in the Wk-4 cardiomyocytes developed a tread-step-tread layout, which typically mimics adult human heart structures, which further confirms the maturation of cardiomyocytes at Wk-4.

Additionally, time-dependent changes in gene expression were analyzed at the molecular level by real-time PCR. We assayed the expression levels of pluripotency markers and cardiac transcription factors to validate differentiation and to verify the cardiac lineage of the hiPSC-CMs. The decreased expression levels of pluripotency markers MYC and SOX2 in Wk-4 hiPSC-CMs indicated their terminal differentiation state. Nonetheless, the increased OCT4 expression in Wk-4 hiPSC-CMs was unexpected. Interestingly, Zangrossi *et al*. detected OCT4 mRNA and protein in terminally differentiated cells, peripheral blood mononuclear cells^44^. Additionally, Zhao *et al*. demonstrated an interaction between OCT4 and CDK1 that inactivated CDK1 *in vitro* to prevent mitotic entry^45^. Furthermore, we detected the decreased expression of SOX-2, which is a downstream transcriptional target of OCT4. Collectively, these findings could account for the expression seen in Wk-4 cells. The increased expression of MEF2C with time in culture and steady expression of NKX2.5 and GATA4 verified the cardiac lineage of these cells. Expression of both pluripotency markers and cardiac transcriptions factors was decreased in NF adult human heart tissue. This may be due to the heterogeneous nature of different cell types present in heart tissue or their long-standing terminal differentiation state. In addition, we then compared the expression levels of contractile genes. Expression of all of the contractile genes we assayed increased over time in culture, which correlates with the functional maturation, metabolic maturation, and observed ultrastructural changes showing more compact and organized sarcomeres. Alternatively, the increased expression of MYL2, TNNT2, and TNNI3 in Wk-4 were in accord with NF adult human heart tissue. Connexin-43, encoded by GJA1, forms gap junctions to couple cardiomyocytes to facilitate electrical conduction and synchronize contraction^46^. The expression of GJA1 was higher in Wk-4 than Wk-1 and Wk-2 hiPSC-CMs. NF adult human heart GJA1 expression was significantly decreased as compared to Wk-1 cells, likely due to these connections already being well-established in the tissue. Importantly, cardiomyocytes need to control the storage and release of calcium for normal contraction-relaxation cycling^47^. Calsequestrin 2, encoded by CASQ2, acts as a Ca^2+^ sponge in the sarcoplasmic reticulum lumen^48^. Additionally, calsequestrin 2 interacts with the Ca^2+^ release channel ryanodine receptor 2, encoded by RYR2^47^. The increased expression of RYR2, CASQ2, CACN1AC, KCNJ2, KCNQ1, and SCN5A in Wk-4 hiPSC-CMs as compared to Wk-1 cardiomyocytes, further strengthens our findings demonstrated by calcium signaling experiments. Increased expression of these genes provides the molecular basis for the observed functional and structural changes seen in Wk-4 hiPSC-CMs. The expression level of genes in Wk-4 hiPSC-CMs often trended towards those in the adult heart, but not always. These discrepancies are likely due to the heterogeneous population of cells in adult human heart tissue.

Cell cycle-related genes play a crucial role in cardiomyocyte maturation and their proliferative potential decreases as they exit the cell cycle^49–52^. Increasing cyclinG1 expression parallels the end of proliferation and promotes the polyploidy of cells by promoting DNA synthesis and inhibiting cytokinesis^53^. In mice, expression of the CDK inhibitor p21^54^ decreases during mid-gestation to birth and peaks at postnatal day 5 to promote cell cycle exit and produce binucleated cells^49^. We observed increased expression of both genes in Wk-4 hiPSC-CMs. A study of fetal/neonatal and adult mouse hearts identified CDK1 and cyclin B1 as top candidates that promote cardiomyocyte proliferation^52^, thus maintaining them in the cell cycle. Therefore, decreased expression of cyclinB1 seen in Wk-4 cells is further indicative of cell cycle exit. Overall, our results justify the maturation of cardiomyocytes as they exit the cell cycle, become binucleated, and exhibit decreased proliferation.

The Lethal-7 (let-7) family of miRNAs were the first miRNAs discovered in humans^55^. They play a role in tumor suppression, CVD, heart development, and cardiovascular differentiation^56,57^. They also have a role in the maturation of hESC-derived cardiomyocytes^58^. In this study, Wk-4 hiPSC-CMs showed that five let-7 family miRNAs (let−7f−5p, miR−98−5p, let−7b−5p, let−7d−5p, and let−7i−5p) were upregulated as compared to Wk-1 hiPSC-CMs, which supports their role in maturation as reported earlier^58^. Several studies have shown that miR−455−5p is upregulated during trophoblast differentiation or under hypoxic conditions^59,60^. The 4-fold upregulation of miR−455−5p in our study indicated its promising role during cardiomyocyte differentiation. Another miRNA, miR−422a, whose upregulation is associated with the onset of stroke, was down-regulated in Wk-4 hiPSC-CMs as compared to Wk-1 hiPSC-CMs^61,62^. The heart exclusively expresses miR−208b-3p, which regulates the myosin heavy chain switch, was upregulated in Wk-4 hiPSC-CMs^63,64^. miR−4443 and miR−1268a, which are upregulated in diabetes and also associated with breast cancer malignancy and hepatocellular carcinoma, were downregulated in Wk-4 hiPSC-CMs^65–67^. Several reports have shown that both miR−15a−5p and miR−32−5p are tumor repressors and induce apoptosis in chronic lymphocytic leukemia^57,68,69^. Further studies are needed to understand the exact role of these above-mentioned miRNAs in cardiomyocyte maturation.

## Conclusion

Overall, this study demonstrated improved functional, structural, metabolic and molecular changes in Wk-4 cultured hiPSC-CMs. The assessment of cardiomyocyte function was confirmed by MEA, PIV, and Ca^2+^ cycling, while confocal microscopy confirmed metabolic maturation. Electron microscopy further correlated functional changes and metabolic maturation with ultra-structural changes, which mimicked the structure and organization of NF adult human heart tissue. Furthermore, gene expression and miRNA profiling demonstrated changes at the molecular level, which suggest the possibility of their roles in cardiomyocyte functional maturation. To the best of our knowledge, we are the first to report differential regulation of twelve miRNAs during time-course culture of hiPSC-CMs. Further gain-of-function and loss-of-function studies are needed to clarify the role of these miRNAs in the functional and structural maturation of cardiomyocytes. In summary, the findings from this study demonstrate that hiPSC-CMs are functionally immature and culturing them for a longer term *in vitro* enhances their functional maturation. Furthermore, transplantation of functionally mature cardiomyocytes into the ischemic heart may have a significant clinical impact on cardiac cell transplantation studies in post-MI patients in the future and could improve the functional outcome of the failing hearts.

## Materials and Methods

### Culturing and maintenance of hiPSC-CMs

The hiPSC-CMs were cultured in a maintenance medium at 5% CO_2_ in a humidified atmosphere at 37 °C, as previously described by our lab^70,71^ (Supplemental Fig. S5A). The analysis showed that 90.6% of the population of hiPSC-CMs was cardiac troponin T-positive (Supplemental Fig. S5B) while immunostaining confirmed the protein expression of pluripotency and cardiac markers (Supplemental Fig. S6).

### Analysis of cardiac functionality on multielectrode array (MEA) system

The sterilized 6-well MEA plate (cat#M384-tMEA-6W) was coated with fibronectin (50 µg/ml) and incubated at 37 °C for 1 h. Each well contained 64 PEDOT microelectrodes. The hiPSC-CMs (4 × 10^4^ cells/well) were plated according to the manufacturer’s protocol and cultured in maintenance medium at 5% CO_2_ in a humidified atmosphere at 37 °C as previously described by our lab^70,71^. Cells were stabilized for 20 min in the MEA system (Maestro Edge, Axion Biosystem, GA, USA) at 5% CO_2_ and at 37 °C. Then data was recorded using AxIS Navigator™ version 1.4.1.9 and analysis was performed on CiPA™ analysis tool version 2.1.10 (Axion Biosystem, GA, USA). Data are expressed as mean ± SD (n = 4).

### Transmission electron microscopy (TEM)

TEM on hiPSC-CMs was performed as previously described by our lab^71^ with minor modifications described in detail in Supplemental Fig. S3. For TEM on NF-adult human heart, sections of freshly isolated human left ventricular myocardial tissue were cut into thin (~2–3 mm) slices and processed for TEM according to standard protocol^72,73^ with minor modifications described in detail in Supplemental Fig. S3.

### Assessment of calcium cycling in hiPSC-CMs

The calcium cycling in hiPSC-CMs was measured with a confocal microscope as described earlier^71,74,75^. In brief, hiPSC-CMs were cultured on fibronectin (50 µg/ml) coated sterile glass coverslips. At the end of each time point cells were incubated in low Ca^2+^ solution (140 mM NaCl, 5.4 mM KCl, 0.5 mM CaCl_2_, 0.5 mM MgCl_2_, 10 mM HEPES, and 5.6 mM glucose, pH 7.4) supplemented with 8 μmol/L fluo‐3 AM (Invitrogen, Carlsbad, CA) for 25 minutes at room temperature. Then the dye was washed out and cells were stimulated at 0.5 Hz with a pair of platinum electrodes in an external solution containing 140 mM NaCl, 5.4 mM KCl, 2.0 mM CaCl_2_, 0.5 mM MgCl_2_, 10 mM HEPES, and 5.6 mM glucose at pH 7.4. Fluo‐3 was excited with the 488 nm line of an argon laser and emission was collected at 500 to 600 nm in the line-scan mode of the confocal microscope (Olympus FLUOVIEW FV1000). Following non-cell background fluorescence subtraction, the fluorescence signal was spatially averaged (excluding non-cell areas) and presented as *F/F*_0_ or *ΔF/F*_0_ *(F − F*_0_*/F*_0_*)*, where *F* is the fluorescence at time *t* and *F*_0_ represents the signal measured at the steady-state level established after cessation of electrical stimulation.

### Assessment of metabolic maturity by confocal microscopy in hiPSC-CMs

To analyze the metabolic maturity of hiPSC-CMs, immunofluorescence microscopy was performed using fatty acid oxidation assay kit (Abcam, MA, USA, Cat # 118183) according to the manufacturer’s protocol. Confocal microscopy (Olympus FV 1000 spectral, Olympus Corporation, PA, USA) was performed to visualize the cells. 12-bit images were captured and image depth was measured and averaged across 4 fields per group using the Olympus FLUOVIEW Ver. 4.2a Viewer.

### Characterization of spontaneous contractile activity in hiPSC-CMs

We have performed a stage analysis on the image sequences, described in detail in Supplemental Fig S1. First, a motion pattern (velocity field) captured on a pair of images was extracted using the optical flow/PIV method described by Zamir *et al*.^76^ and Aleksandrova *et al*.^77^. As described by Rajasingh *et al*.^8,78^, each consecutive frame pair of a 30 sec long video recording was analyzed to identify suitable reference frames when the movement is minimal and thus cardiomyocytes are in a relaxed state. In the second set of PIV calculations, we compared each frame to a reference frame and thus obtained beat displacement maps. The spatially resolved beat displacement maps were further averaged around selected locations yielding beat patterns. To distinguish active contractility to passive (elastic) deformations, we calculated convergence fields as described by Czirok *et al*.^79^. To extract the frequency of cardiomyocyte beating activity, beat patterns were subjected to Fourier analysis, and the dominant beat frequencies were visualized on power spectrum plots.

### Procurement of human heart samples

The studies on human heart tissue were performed under the guidelines of the Declaration of Helsinki, with oversight by the Institutional Review Boards at The Ohio State University (protocol no. 2012H0197). All patients provided informed consent per IRB guidelines for this study. NF adult human hearts were obtained in collaboration with the Lifeline of Ohio Organ Procurement program from organ donors without diagnosed heart failure whose hearts were not suitable for transplantation. These donors died from causes other than heart failure. Hearts were removed from the patients/donors and immediately submerged in ice-cold cardioplegic solution containing 110 mM NaCl, 16 mM KCl, 16 mM MgCl_2_, 10 mM NaHCO_3_, and 0.5 mM CaCl_2_. Tissues were then flash-frozen in liquid nitrogen and stored at −80 °C until used for this study. Biopsies from the free wall of the left ventricle were used in this study.

### Human heart tissue homogenization

NF adult human heart tissue was chopped into small pieces by using a sharp surgical blade. Then heart tissue was kept in a cleaned mortar and pestle for homogenization. Once fully ground, 1 ml Trizol reagent was added to it. The tissue homogenate was transferred and further homogenized using Dounce homogenizer for approximately 2–3 minutes. Then the tissue homogenate was transferred into a 1.5 ml centrifuge tube and centrifuged at 13,000 rpm for 15 minutes. The supernatant was collected in a 15 ml centrifuge for RNA isolation and isolated as described below.

### RNA isolation

hiPSC-CMs cultured at different weeks were washed with PBS, 1 ml Trizol added to cells and the lysate was collected. Then the lysate was centrifuged at 13,000 rpm for 15 minutes. The supernatant was collected and an equal volume of ethanol (100%) was added and mixed thoroughly. RNA was then extracted using the Direct-zol RNA Miniprep kit as per the manufacturer’s protocol (Zymo Research, Irvine, CA). RNA estimation was performed on NanoDrop 2000 spectrophotometer (Thermo Fisher, NY, USA) and stored at −80 °C for further analysis.

### cDNA synthesis

Synthesis of cDNA was performed using Qiagen RT2 First Strand cDNA synthesis kit (cat # 330404) according to the manufacturer’s protocol but with synthesis at 37 °C for 1 hour. 200 ng of input RNA was used for all reactions. For cell cycle gene analysis cDNA was synthesized using the High Capacity cDNA Reverse Transcription Kit (cat # 4368814, Applied Biosystems, CA) according to the manufacturer’s protocol with 480 ng of input RNA for all reactions.

### Real-Time quantitative PCR

RT-qPCR was performed using Qiagen RT2 SYBR green ROX qPCR Mastermix (Cat # 330523) according to the manufacturer’s protocol. The RT2 Profiler PCR array was custom designed to analyze the expression of cardiac maturity markers. The reaction was performed on a QuantStudio 3 (Applied Biosystems, USA) using QuantStudio design and analysis software V.1.4.1. Gene expression was normalized to the geometric mean of housekeeping genes β-Actin and PGK1 then calculated relative to Wk-1 expression using the 2^−ΔΔCt^ method^80^. Data is expressed as mean ± SD, n = 3 for hiPSC-CMs, n = 9 for NF adult human heart samples.

To assess cell cycle gene expression, RT-qPCR was performed using TaqMan Universal qPCR Master Mix (4304437, Applied Biosystems, CA). The reaction was run as above per cycle instructions for the master mix. TaqMan assays included: cyclinG1 (Hs00171112_m1), p21 (Hs99999142_m1), cyclinB1 (Hs01030099_m1), and β-actin (4333762 T) (ThermoFisher, MA). Gene expression was normalized to β-actin and calculated relative to Wk1- using the 2^−ΔΔCt^ method, data represents mean ± SD, n = 3 for hiPSC-CMs, n = 9 for NF adult human heart samples.

### miRNA analysis

miRNA analysis for hiPSC-CMs was performed in quadruplet samples according to manufacturers’ protocol (NanoString Technologies, Inc. Seattle, USA) by using nCounter Human v3 miRNA Expression Assay Kit (Cat# GXA-MIR3-12). Briefly, 100 ng of total RNA was annealed with multiplexed DNA tags (miR-tag) and bridges target specifics. Mature miRNAs were bound to specific miR-tags using a Ligase enzyme and all the tags in excess were removed by enzyme clean-up step. The tagged miRNA product was diluted 5 times and 5 µl was combined with 20 µl of Reported Probes in hybridization buffer and 5 µl of Capture probes. The overnight hybridization (16 to 20 hours) at 65 °C allowed sequence-specific probes to complex with targets. Excess probes were removed using two-step magnetic bead-based purification on an automated fluidic handling system (nCounter Prep Station) and target/probe complexes were immobilized on the cartridge for data collection. The nCounter Digital Analyzer collected the data by taking images of immobilized fluorescent reporters in the sample cartridge with a CCD. For each cartridge, 325 fields of view were performed.

### Statistical analysis

Data were analyzed for assumptions of normality and equal variance. If both assumptions were met then Student’s t-test, two-tailed at alpha = 0.05, was used to compare between groups. If the equal variance assumption was not met, then a Welch’s t-test, two-tailed at alpha = 0.05 was used to compare between groups. If neither assumption was met, then the Mann-Whitney Rank Sum test, two-tailed with alpha = 0.05, was used to compare between groups. All values were expressed as Mean ± SD, a value of p < 0.05 was considered to be statistically significant.

## Supplementary information

Supplemental data
