# Supplementary material for: Assessment of temporal functional changes and miRNA profiling of human iPSC-derived cardiomyocytes
Source: Sci Rep. 2019 Sep 12;9:13188. doi: 10.1038/s41598-019-49653-5 (PMC6742647; doi:10.1038/s41598-019-49653-5)
Supplement: Supplementary file 1 — Supplemental data [file 41598_2019_49653_MOESM1_ESM.pdf]

Assessment of temporal functional changes and miRNA profiling of human iPSC-derived cardiomyocytes

Naresh Kumar[1], Julie A. Dougherty[1], Heather R. Manring[2], Ibrahim Elmadbouh[1], Muhamad Mergaye[1], Andras Czirok[5], Dona Greta Isai[5], Andriy E. Belevych[2], Lianbo Yu[3], Paul M.L. Janssen[2], Paolo Fadda[4], Sandor Gyorke[2], Maegen A. Ackermann[2], Mark G. Angelos[1], Mahmood Khan[1,2,*]

[1]Department of Emergency Medicine, Dorothy M. Davis Heart Lung and Research Institute, The Ohio State University Wexner Medical Center, Columbus, OH, USA.

[2]Department of Physiology and Cell Biology, The Ohio State University Wexner Medical Center, Columbus, OH, USA.

[3]Center for Biostatistics, College of Medicine, The Ohio State University Wexner Medical Center, Columbus, OH, USA.

[4]Comprehensive Cancer Center, The Ohio State University Wexner Medical Center, Columbus, OH, USA.

[5]Department of Anatomy and Cell Biology, University of Kansas Medical Center, Kansas City, KS, USA.

Short Title: Functional maturation of cardiomyocytes

Keywords: Human induced pluripotent stem cell-derived cardiomyocytes, Myocardial Infarction, Cardiovascular disease, miRNA, Multielectrode array (MEA)

***Corresponding Author:**

Mahmood Khan, M. Pharm, Ph.D., FAHA

Department of Emergency Medicine

Department of Physiology and Cell Biology

Davis Heart and Lung Research Institute

The Ohio State University Wexner Medical Center

473 W. 12th Avenue, Columbus, OH 43210

E-mail: mahmood.khan@osumc.edu

Methods for Characterization of spontaneous contractile activity

Image analysis

Cross-correlation analysis of images. A motion pattern (velocity field) captured on a pair of images was extracted using the method described by Zamir *et al*.,[1] and Aleksandrova *et al*.,[2]. Briefly, the first image was divided into 64 pixels wide overlapping square tiles. The displacement of each tile was determined by the following cross-correlation analysis. The second image was scanned by shifting an equally sized window, and for each location, the pattern within the window was compared with the tile of the first image. The scanned area was centered at the position of the tile and extended for 64 pixels in each direction. The similarity of two image tiles was quantified by the value of their cross-correlation: the pixel-by-pixel sum of the product $a(x)b(x)$ where $a(x)$ and $b(x)$ denote the brightness of corresponding pixels x within the original tile of the first image and within the window positioned on the second image, respectively. This procedure yields a location which exhibits the most similar pattern to the pattern within the tile of the first image. The difference between the locations of the first tile and its most similar replica in the second image yields a displacement vector. Repeating this procedure for each tile of the first image, we thus derive a motion vector estimate for various image locations. This estimate of movement is then interpolated and de-noised by a thin-plate spline fit, yielding our coarse displacement field. The coarse estimate was used to construct a second, higher resolution displacement field. In this second step, the cross-correlation search for pattern similarity was repeated with tiles that were only 32 pixels wide and in a much smaller search area allowing only for 4-pixel displacements around the location predicted by the coarse displacement field.

Beat patterns

As described by Rajasingh *et al*.,[3,4] each consecutive frame pair of a 30 sec long video recording was analyzed by our cross-correlation/PIV technique. The frame-by-frame displacements were used to identify suitable reference frames that correspond to moments when movement is minimal and thus cardiomyocytes are in a relaxed state. In the second set of cross-correlation calculations, we then compared each frame to a reference frame and thus obtained beat displacement maps $d\ (t, x)$, which estimate for each time point t and location x the total movement (magnitude and directionality) relative to a resting (contraction-free) state. The spatially resolved beat displacement maps were further averaged around selected locations (e.g., within a 100-pixel radius), which procedure yielded beat patterns $D\ (t)$. Thus, $D\ (t)$ is the spatial average of the magnitudes $|d\ (t, x)|$ for each time point t. Beat patterns consist of a sequence of peaks, each corresponding to a contractile event. A single peak of the $D\ (t)$ beat pattern typically rises suddenly and diminishes approximately as an exponential function.

To distinguish active contractility to passive (elastic) deformations, we calculated convergence fields as described by Czirok *et al*.,[5]. The divergence of a vector field in two dimensions gives the net flux passing through the perimeter of a small area. A positive or negative divergence value thus indicates the presence of a source or sink at that position, respectively. Thus, contracting cardiomyocytes display negative divergence, which we termed convergence Czirok *et al*.,[5]. Convergence fields are established from the $d\ (t, x)$ beat displacement maps as a filtered combination of numerical spatial derivatives.

Statistical characterization of beat patterns

To extract the frequency of cardiomyocyte beating activity, $D(t)$ beat patterns were subjected to Fourier analysis, and the dominant beat frequencies were visualized on power spectrum plots. Power densities were obtained as the magnitudes of the squared Fourier spectra, and are used to indicate periodicity within the signal in the form of peaks at the corresponding frequencies. When the analyzed signal is not pure sine wave, harmonics may appear at integer multiples of the fundamental frequency f (at $2f$, $3f$, etc.). The magnitude of a peak is related to the amplitude of the signal component oscillating with the corresponding frequency.

Average waveform

To establish the typical waveform $w(t)$ of the contraction peaks in the beat pattern $D(t)$, we first identified the local maxima $\{m_k\}$ in the time series. The peak around the second maximum m_2 was used as an initial estimate of the waveform: $w(t) = D(t-m_2)$ for -2 sec < t < 2 sec. Then, each peak k was translated with an offset o_k so that its overlap with the current average waveform was maximal, i.e., the sum of their point-by-point differences $|w(t) - D(t- m_k + o_k)|$ were minimal. The peak, at its optimal position, was then included in the calculation of the next estimate of the average waveform: $w(t) \leftarrow [(k-1)\, w(t) + D(t - m_k + o_k)]/k$.

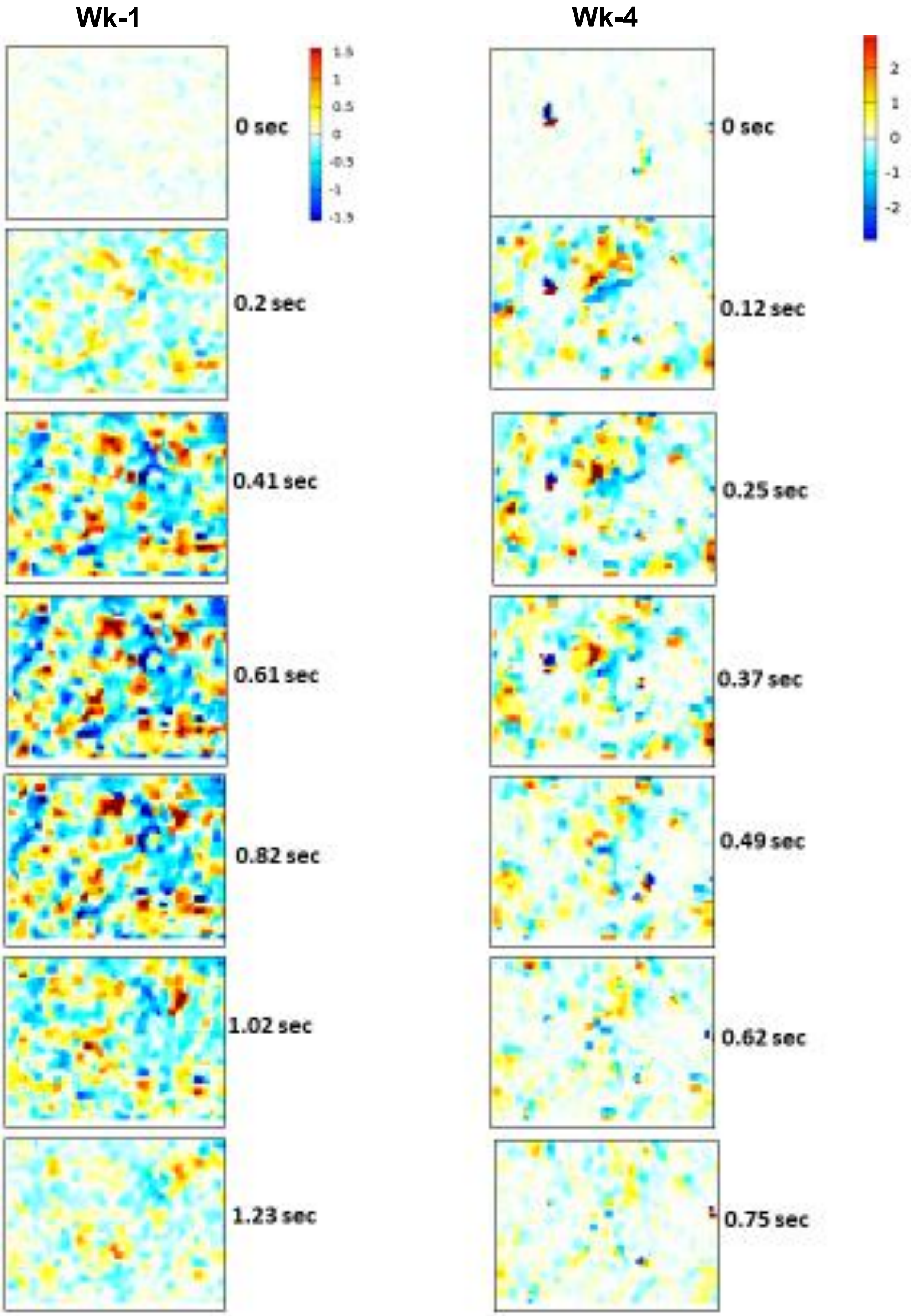

Wk-1
Wk-4
0 sec
0.2 sec
0.41 sec
0.61 sec
0.82 sec
1.02 sec
1.23 sec
0 sec
0.12 sec
0.25 sec
0.37 sec
0.49 sec
0.62 sec
0.75 sec

Supplemental Figure 1: Time-resolved contractility maps characterize representative contractile events in Wk-1 (left) and Wk-4 hiPSC-CMs (right) cultures. Contractile behavior is indicated by warmer colors, while cooler colors indicate local expansion of the cell collective. Irrespective of the age of cultures, the onset of contraction at the spatially disjunction contractile centers is synchronous. Contractility is given in units of 1/sec.

Immunostaining and assessment of metabolic maturity by confocal microscopy

To analyze the metabolic maturity of hiPSC-CMs, immunofluorescence microscopy was performed using fatty acid oxidation assay kit (Abcam, MA, USA, Cat # 118183) according to the manufacturer's protocol. Confocal microscopy (Olympus FV 1000 spectral, Olympus Corporation, PA, USA) was performed to visualize the cells.

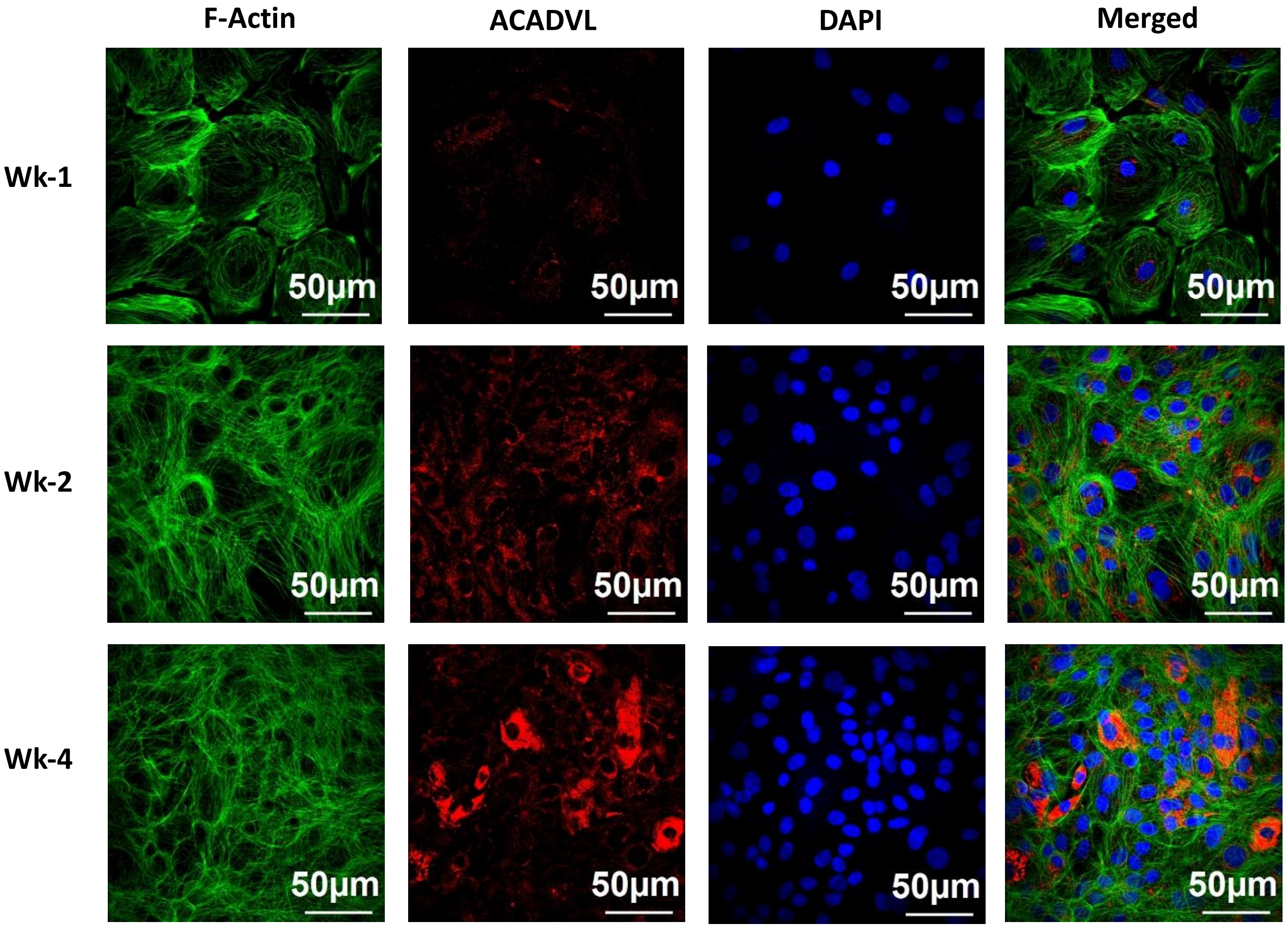

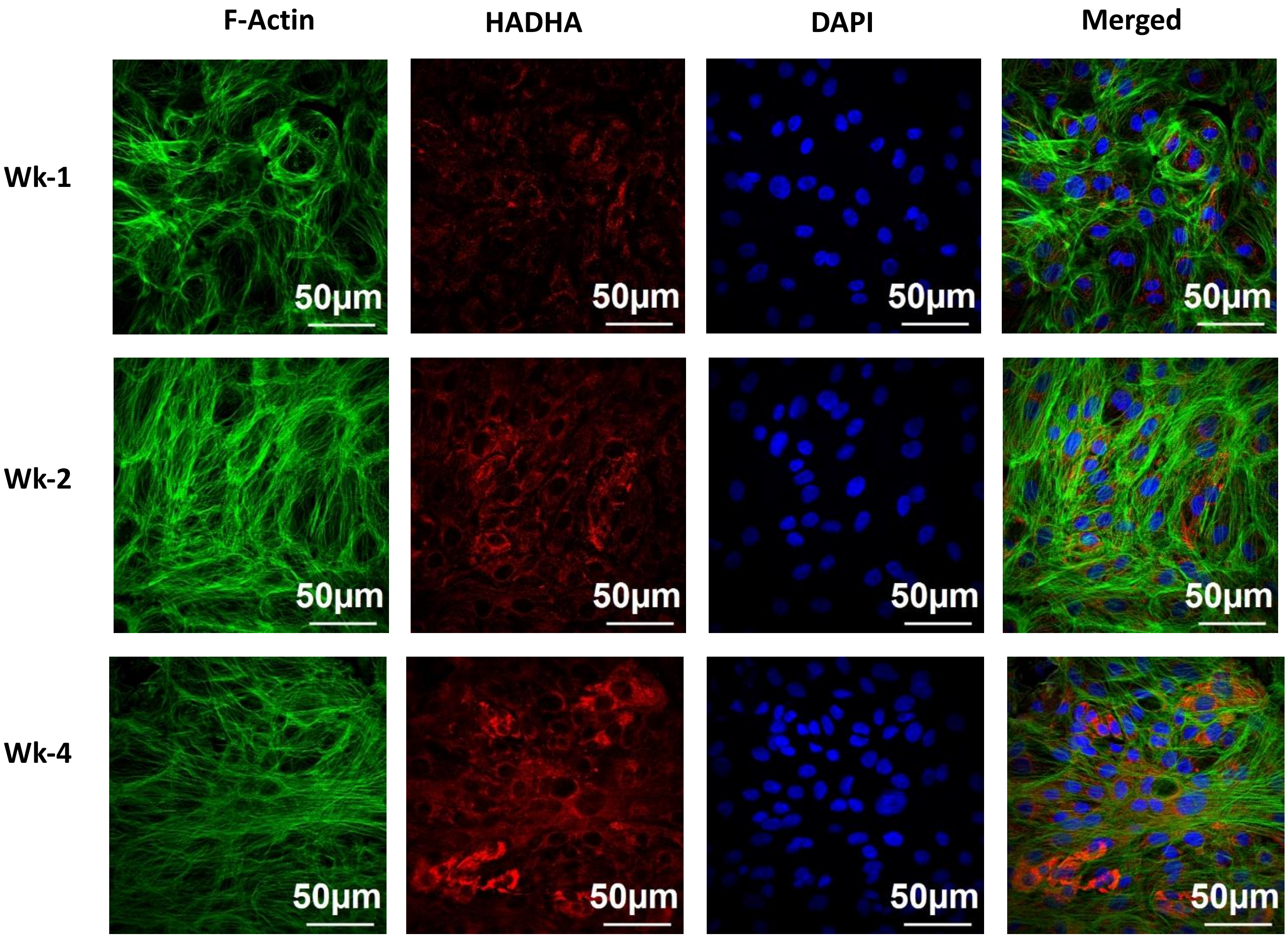

Supplemental Figure 2: Immunofluorescence microscopy showing metabolic maturation in prolonged cultured hiPSC-CMs. Cells were cultured for Wk-1, Wk-2, and Wk-4 and then immunostained with antibodies against two vital enzymes of FAO pathways, acyl-CoA dehydrogenase very long chain, (ACADVL, EC 1.3.99.13) and α-subunit of long-chain 3-hydroxyacyl coenzyme A dehydrogenase (HADHA, EC 1.1.1.211). The confocal microscopy showed that the expression of ACADVL and HADHA was significantly ($p<0.05$) high in Wk-4 as compared to Wk-1 and Wk-2 hiPSC-CMs.

Transmission Electron Microscopy (TEM) on NF adult human heart

Sections of freshly isolated human left ventricular myocardial tissue were cut into thin (~2-3mm) slices and processed for Transmission Electron Microscopy (TEM) according to standard protocol[6,7], with minor modifications. Specifically, thin slices were immediately fixed by immersion fixation in 2% glutaraldehyde, 4% paraformaldehyde in 0.1M phosphate buffer pH 7.4 containing 0.1M sucrose for 4-6 at 4˚C. Longitudinal sections were cut from the thinly sliced myocardium into appropriately sized pieces (~1-2 mm^3) and incubated in the above fixative for an additional 1 hour. Samples were washed five times in 0.1M phosphate buffer pH 7.4 containing 0.1M sucrose, post-fixed for 2 hours in 1% osmium tetroxide in 0.1M phosphate buffer pH 7.4, washed in distilled water followed by a 1 hour en bloc stain with ethanolic 2% uranyl acetate. Samples were dehydrated in a graded ethanol series and then transitioned into acetone. A graded acetone:resin (Eponate 12, Ted Pella, Inc.) series was used for tissue infiltration and subsequently samples were embedded in freshly prepared resin in small tapered, flat embedding molds (Ted Pella, Inc.) orientated such that longitudinal sections could be acquired. Blocks were polymerized overnight at 65˚C. Next, blocks were trimmed and semi-thin sections (500 nm) were acquired using a glass knife. Blocks were further trimmed, and 80 nm thin sections were cut using a diamond knife (Diatome) on a Leica EM UC6 ultra-microtome. Ultra-thin sections were collected on 200 mesh copper grids and stained with 1% uranyl acetate and Reynold's lead citrate. Observations were made on a FEI Tecnai G2 Biotwin TEM operating at 80 kV and micrographs captured using an AMT camera.

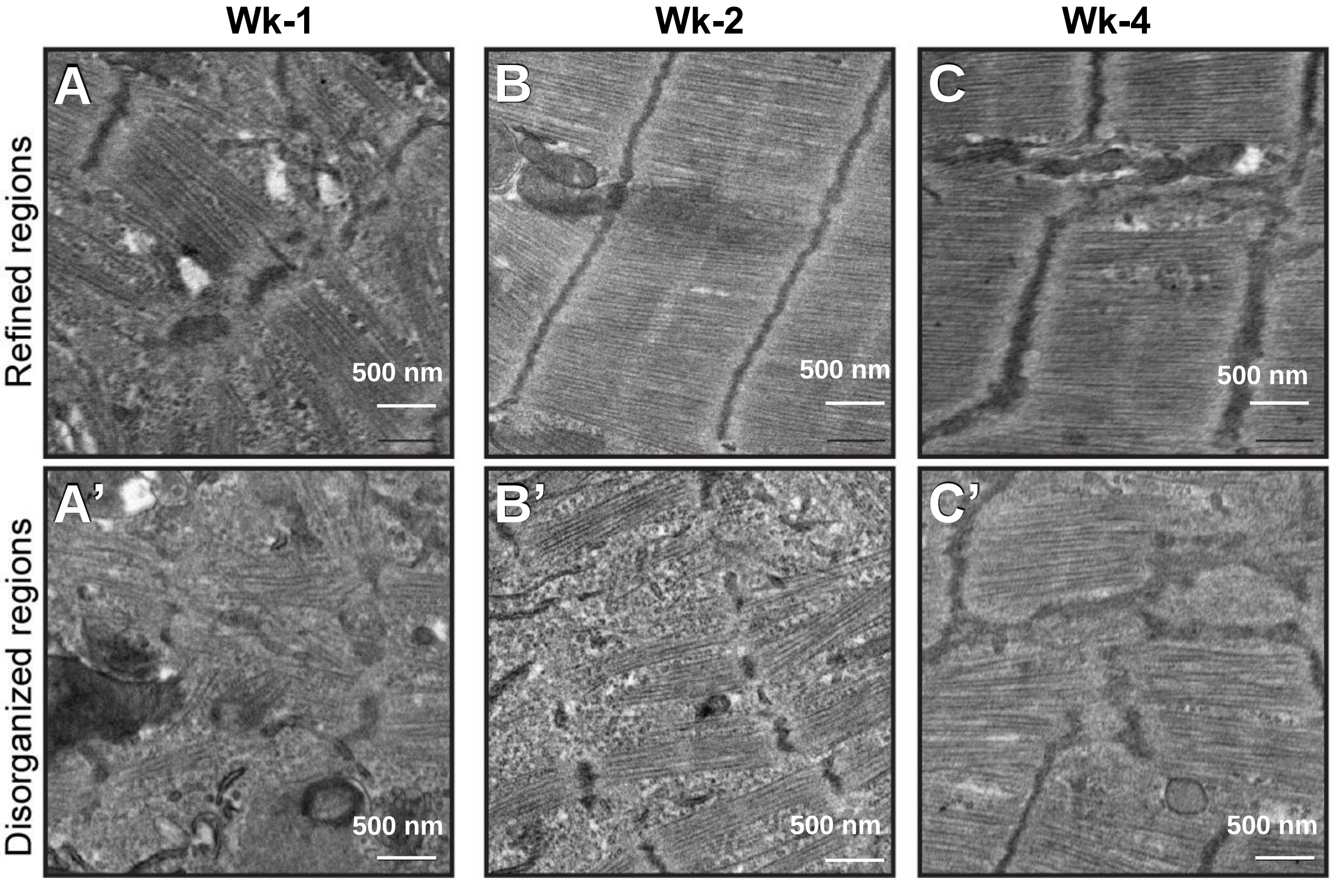

Supplemental Figure 3: **Organization levels vary within hiPSC-CM cultures.** Different regions of Wk-1, Wk-2, and Wk-4 hiPSC-CMs present different overall levels of sarcomere organization and refinement of ultrastructural features. **(A-C)** Representative images of more "refined" regions of Wk-1, Wk-2, and Wk-4 cultured hiPSC-CMs. **(A'-C')** Representative images of more disorganized regions identified in Wk-1, Wk-2, and Wk-4 hiPSC-CM cultures. Scale bar, 500 nm.

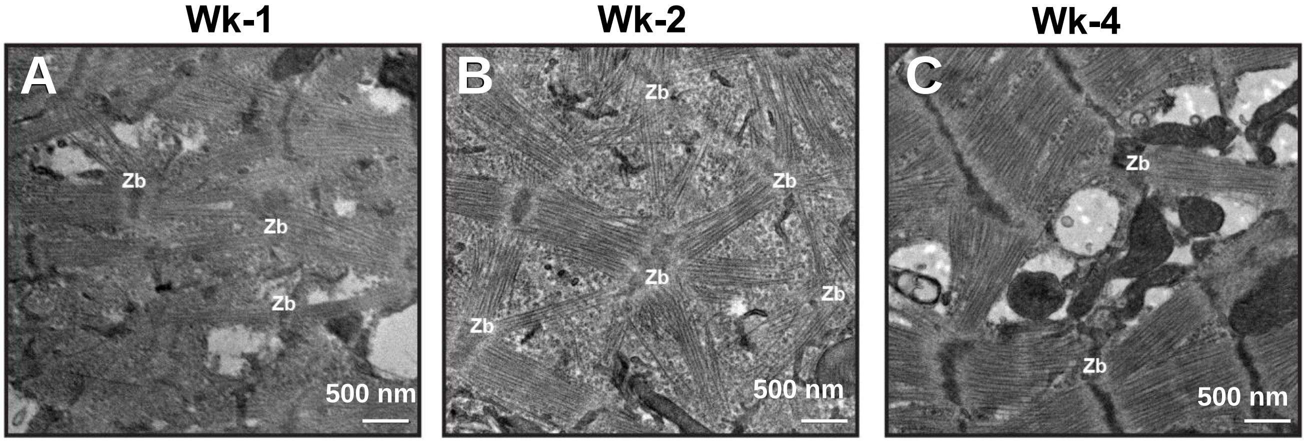

Supplemental Figure 4: Sarcomerogenesis in hiPSC-CMs. The organization and addition of sarcomeres (sarcomerogenesis) is present throughout the four weeks hiPSC-CMs cultured but most prominent at Wk-2. **(A-C)** Organization of myofilaments from Z-bodies (Zb) is seen in Wk-1, Wk-2, and Wk-4 hiPSC-CMs. Wk-2 hiPSC-CMs show large numbers of Z-bodies and “star-like” organization of sarcomeric units. Scale bar, 500 nm.

Culturing and maintenance of hiPSC-CMs

hiPSC-CMs were procured from Cellular Dynamics International (CDI, WI, USA) (Cat# R 1007). The hiPSC-CMs were plated according to the manufacturer's protocol and cultured in maintenance medium at 5% CO_2 in a humidified atmosphere at 37 °C, as previously described by our lab[8,9].

Flow cytometry analysis of hiPSC-CMs

hiPSC-CMs were plated onto a 12-well plate at a density of 0.3 X 10^6 cells/well. Cells were harvested with trypsin. The hiPSC-CMs pellet was suspended in 250 µl of BD staining buffer and centrifuged at 300 *g* for 5 minutes. For fixation/permeabilization, the cell pellet was suspended in BD cytofix/cytoperm buffer and incubated on ice for 30 minutes then washed two times with BD Perm/wash buffer. Then cells were incubated with either Alexa Fluor® 647-conjugated mouse IgG1 κ isotype control (557732, BD Bioscience, CA) or Alexa Fluor® 647-conjugated mouse anti-cardiac troponin T (565744, BD Bioscience, CA) for 1 h on ice. The stained cells were washed twice with BD Perm/wash buffer. Finally, 400 µl of staining buffer was used to resuspend the cells and flow-cytometry analysis was performed on BD™ LSR II using BD FACSDiva™ software (BD Bioscience, CA). The data was analyzed on FlowJo® version 10.4.1 (FlowJo, LLC; Becton, Dickinson & Company,).

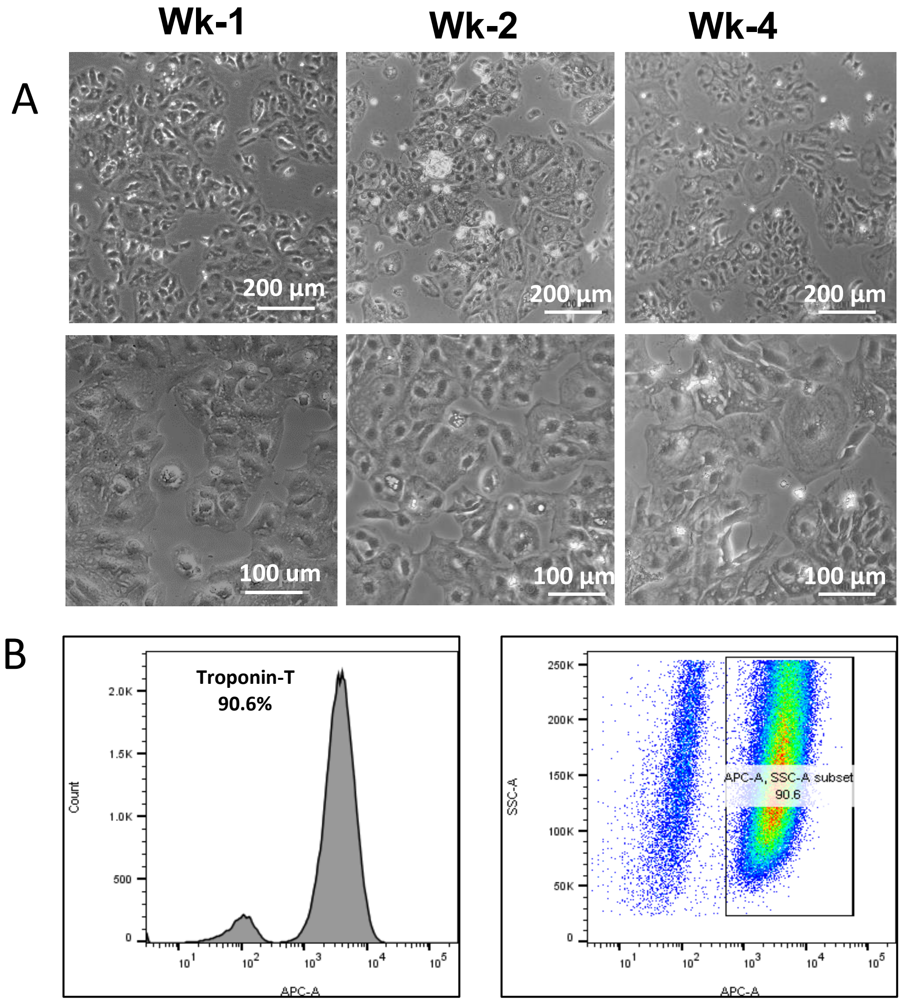

Supplemental Figure 5: Assessment of phenotype and cardiac markers in hiPSC-CMs. (**A**) hiPSC-CMs were cultured for Wk-1, Wk-2, and Wk-4 and phase-contrast images show that cells were healthy in culture during the experiment. (**B**) Flow cytometry analysis of Wk-2 hiPSC-CMs stained for Alexa Fluor® 647-conjugated cardiac troponin T. The analysis showed that 90.6 % of the population of hiPSC-CMs was cardiac troponin T-positive.

Immunostaining and assessment of cardiac maturity markers by confocal microscopy

To analyze the expression of pluripotency and cardiac maturity markers, immunofluorescence microscopy was performed as described earlier [9]. Briefly, hiPSC-CMs were seeded on sterile glass coverslips coated with fibronectin (50 µg/ml) and cultured for different time points (Wk-1, Wk-2, and Wk-4). At the end of each time point, cells were washed with 1X PBS twice and incubated with 4% paraformaldehyde for 10 min at room temperature for fixation. Cells were washed with 1X PBS and blocking (1 hour) was performed in TBS containing 10% normal goat serum and 0.1% Tween-20 to reduce non-specific binding. Cells were then incubated with primary antibodies against Troponin-T (HPA017888, Sigma), Troponin-I (701585 ThermoFisher Sc. USA), Connexin-43 (MAB 3067, Millipore), α-sarcomeric actinin (A7811, Sigma), GATA4 (PA1-102, ThermoFisher Sc. USA), and NKX 2.5 (PA5-49431, ThermoFisher Sc.), for 2 hours at room temperature and then washed with 1X PBS (3 times) for 5 min interval and incubated for 1 hour with corresponding secondary antibodies: anti-mouse (1:5000, Cell Signaling, 4408S,) or anti-rabbit (1:5000, Cell Signaling, 8889S) conjugated with either Texas Red or FITC. NucBlue (R37605, Invitrogen) was used to stain the nucleus. The cells were washed with 1X PBS (3 times for 5 min) and coverslips were mounted on slides with mounting medium (VectaMount, H-5501, Vector laboratories Inc. CA, USA). Confocal microscopy (Olympus FV 1000 spectral, Olympus Corporation, PA, USA) was performed to visualize the cells and image analysis was performed on Olympus FLUOVIEW Ver. 4.2a Viewer.

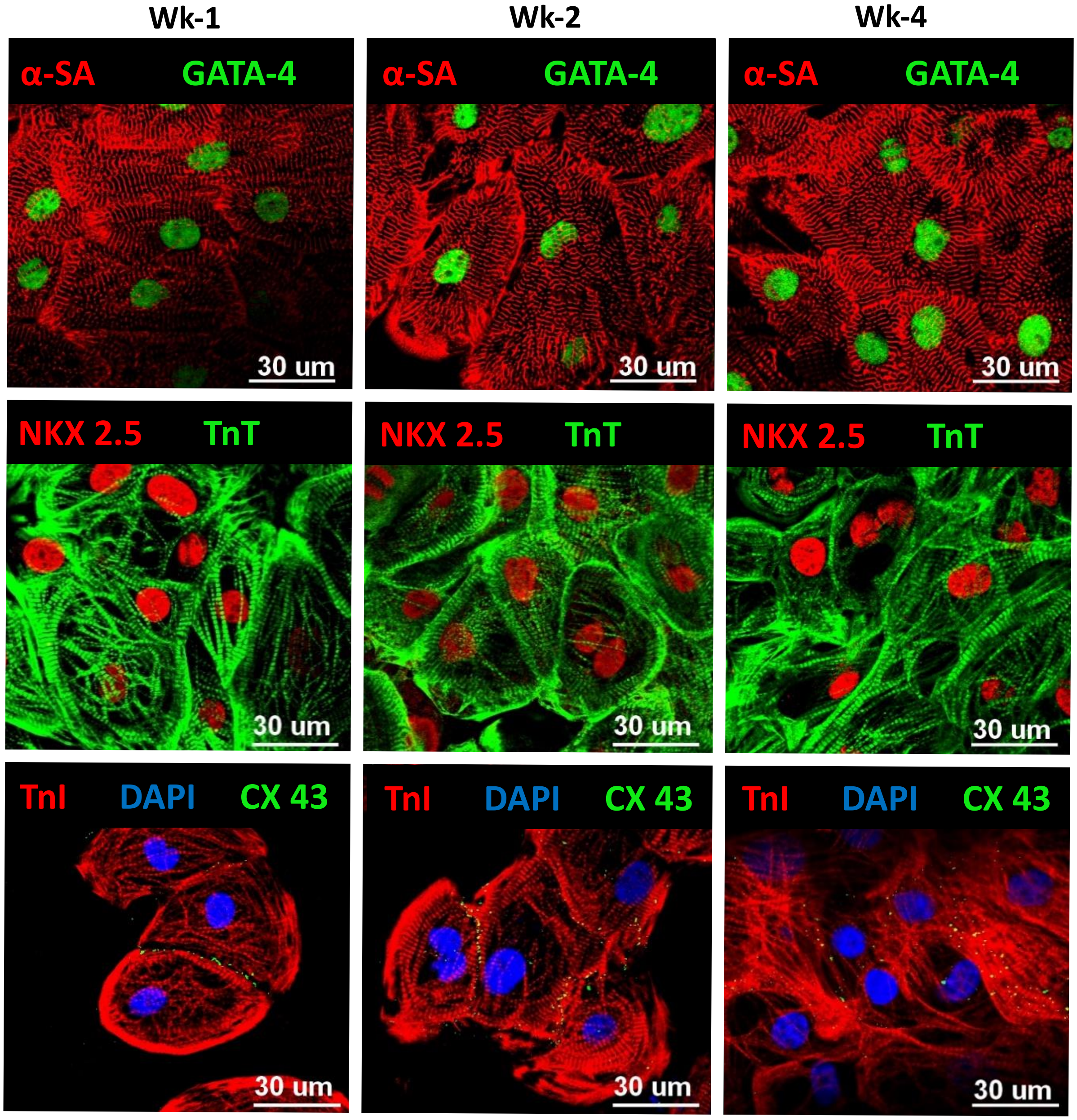

Supplemental Figure 6: Immunofluorescence microscopy showing the expression of cardiac markers in hiPSC-CMs. Cells were cultured for Wk-1, Wk-2, and Wk-4 and then immunostained with antibodies against α-SA, NKX2.5, GATA-4, TNNT2 (TnT), TNNI1 (TnI) and Connexin-43 (CX 43). The analysis showed that hiPSC-CMs expressed all of these cardiac markers over the course of the experiment.

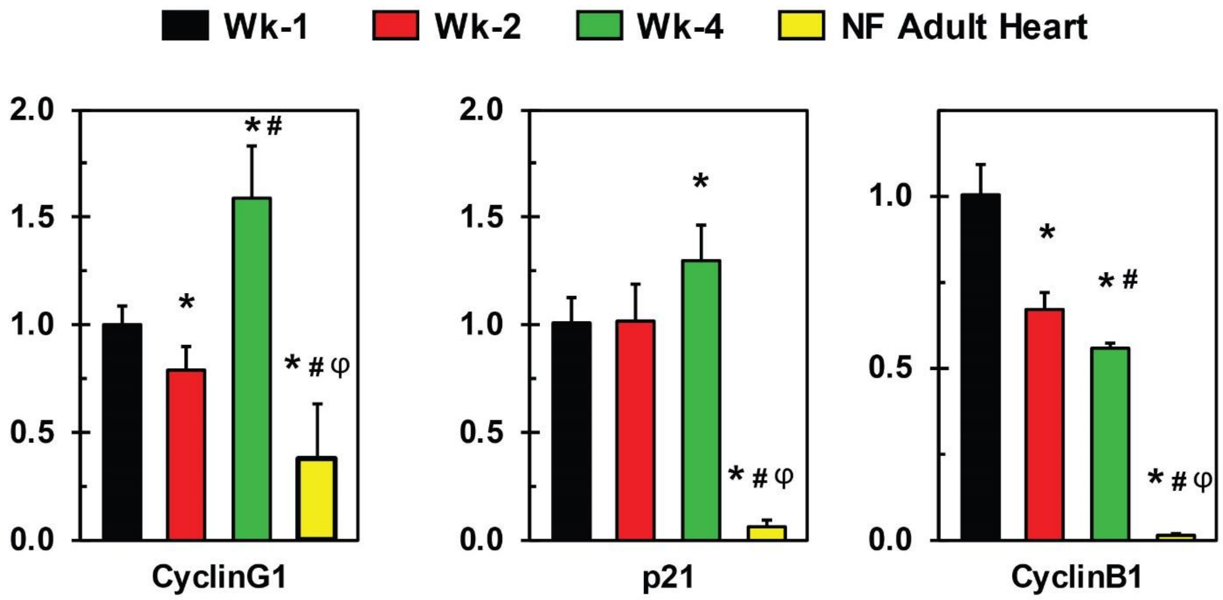

Supplemental Figure 7: RT-qPCR analysis showing the expression of cell cycle genes in hiPSC-CMs. Cells were cultured for Wk-1, Wk-2, and Wk-4 and the results show expression of cell cycle genes. Data expressed as mean±SD, n=4, *p < 0.05 vs Wk-1, #p < 0.05 vs Wk-2, φp<0.05 vs Wk-4

.

References:

1. Zamir, E. A., Czirók, A., Cui, C., Little, C. D. & Rongish, B. J. Mesodermal cell displacements during avian gastrulation are due to both individual cell-autonomous and convective tissue movements. *Proc. Natl. Acad. Sci. U. S. A.* **103**, 19806–11 (2006).
2. Aleksandrova, A. *et al.* Convective tissue movements play a major role in avian endocardial morphogenesis. *Dev. Biol.* **363**, 348–361 (2012).
3. Rajasingh, S. *et al.* Generation of Functional Cardiomyocytes from Efficiently Generated Human iPSCs and a Novel Method of Measuring Contractility. *PLoS One* **10**, e0134093 (2015).
4. Rajasingh, S. *et al.* Manipulation-free cultures of human iPSC-derived cardiomyocytes offer a novel screening method for cardiotoxicity. *Acta Pharmacol. Sin.* (2018). doi:10.1038/aps.2017.183
5. Czirok, A. *et al.* Optical-flow based non-invasive analysis of cardiomyocyte contractility. *Sci. Rep.* **7**, 10404 (2017).
6. Ackermann, M. A. *et al.* TGF-β1 affects cell-cell adhesion in the heart in an NCAM1-dependent mechanism. *J. Mol. Cell. Cardiol.* **112**, 49–57 (2017).
7. Ackermann, M. A. *et al.* Integrity of the network sarcoplasmic reticulum in skeletal muscle requires small ankyrin 1. *J. Cell Sci.* **124**, 3619–30 (2011).
8. Citro, L. *et al.* Comparison of Human Induced Pluripotent Stem-Cell Derived Cardiomyocytes with Human Mesenchymal Stem Cells following Acute Myocardial Infarction. *PLoS One* **9**, e116281 (2014).
9. Khan, M. *et al.* Evaluation of Changes in Morphology and Function of Human Induced Pluripotent Stem Cell Derived Cardiomyocytes (HiPSC-CMs) Cultured on an Aligned-Nanofiber Cardiac Patch. *PLoS One* **10**, e0126338 (2015).
